# Author Correction: A high-conductivity *n*-type polymeric ink for printed electronics

**DOI:** 10.1038/s41467-022-29482-3

**Published:** 2022-03-29

**Authors:** Chi-Yuan Yang, Marc-Antoine Stoeckel, Tero-Petri Ruoko, Han-Yan Wu, Xianjie Liu, Nagesh B. Kolhe, Ziang Wu, Yuttapoom Puttisong, Chiara Musumeci, Matteo Massetti, Hengda Sun, Kai Xu, Deyu Tu, Weimin M. Chen, Han Young Woo, Mats Fahlman, Samson A. Jenekhe, Magnus Berggren, Simone Fabiano

**Affiliations:** 1 Laboratory of Organic Electronics, Department of Science and Technology, Linköping University, Norrköping, Sweden; 2 Department of Chemical Engineering and Department of Chemistry, University of Washington, Seattle, WA USA; 3 Department of Chemistry, College of Science, Korea University, Seoul, 136-713 Republic of Korea; 4 Department of Physics, Chemistry and Biology, Linköping University, Linköping, Sweden; 5 Wallenberg Wood Science Center, Linköping University, Norrköping, Sweden; 6 n-Ink AB, 58330 Linköping, Sweden

**Keywords:** Electronic materials, Conjugated polymers, Electronic devices

Correction to: *Nature Communications* 10.1038/s41467-021-22528-y, published online 21 April 2021.

The original version of this Article omitted the following statement from the Acknowledgements:

‘We acknowledge MAX IV Laboratory for time on Beamline SPECIES-APXPS under Proposal 20200356. Research conducted at MAX IV, a Swedish national user facility, is supported by the Swedish Research council under contract 2018-07152, the Swedish Governmental Agency for Innovation Systems under contract 2018-04969, and Formas under contract 2019-02496.’

This has been added to the Acknowledgements in both the PDF and HTML versions of the Article.

