# Supplementary material for: A high-conductivity n-type polymeric ink for printed electronics
Source: Nat Commun. 2021 Apr 21;12:2354. doi: 10.1038/s41467-021-22528-y (PMC8060302; doi:10.1038/s41467-021-22528-y)
Supplement: Supplementary file 1 — Supplementary Information [file 41467_2021_22528_MOESM1_ESM.pdf]

Supplementary Information

A high-conductivity n-type polymeric ink for printed electronics

C.-Y. Yang et al.

Contents:

Supplementary Figures 1-38

Supplementary Tables 1-6

Supplementary References

Supplementary Figures 1-38

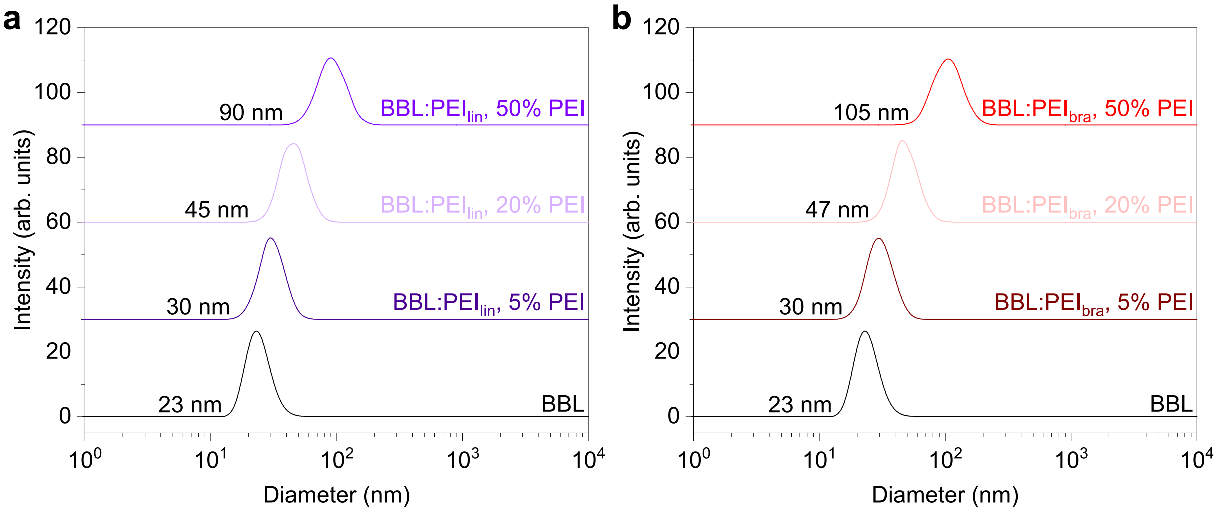

Supplementary Figure 1 | Ink particle size distribution. a-b Dynamic light scattering (DLS) analysis of BBL:PEI_{lin} (**a**) and BBL:PEI_{bra} (**b**) ethanol-based ink.

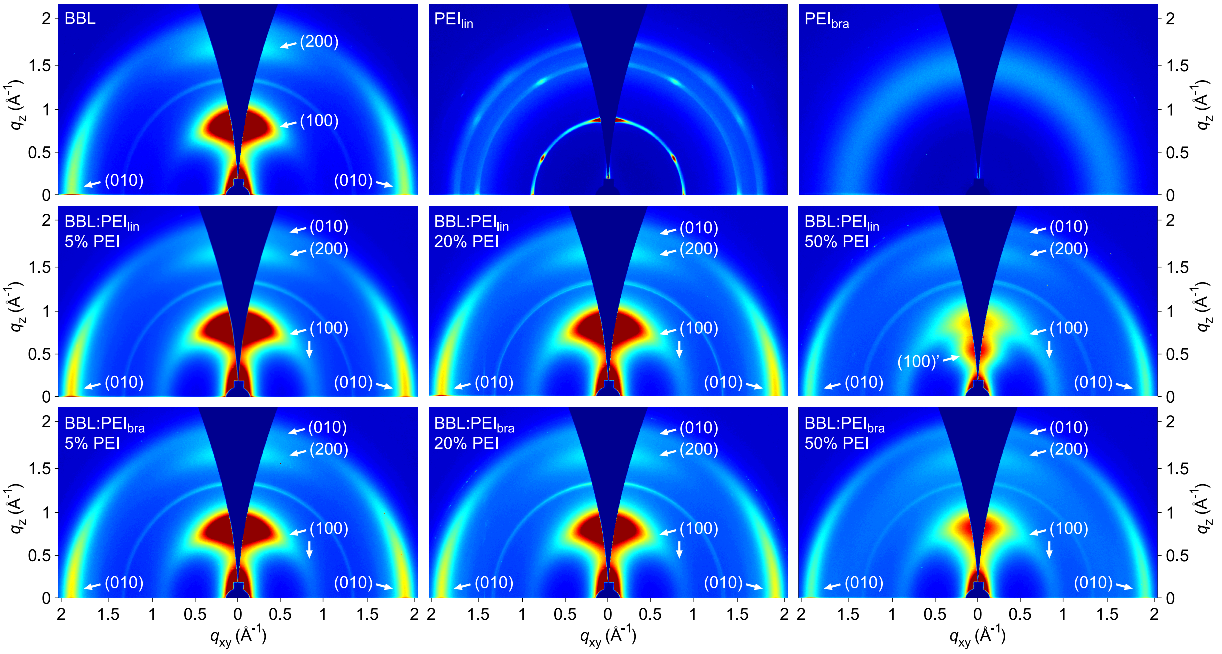

Supplementary Figure 2 | 2D GIWAXS analysis. 2D grazing incidence wide angle X-ray scattering (GIWAXS) patterns of pristine BBL, PEI_{lin}, PEI_{bra}, and BBL:PEI with 5, 20, and 50 wt-% PEI content.

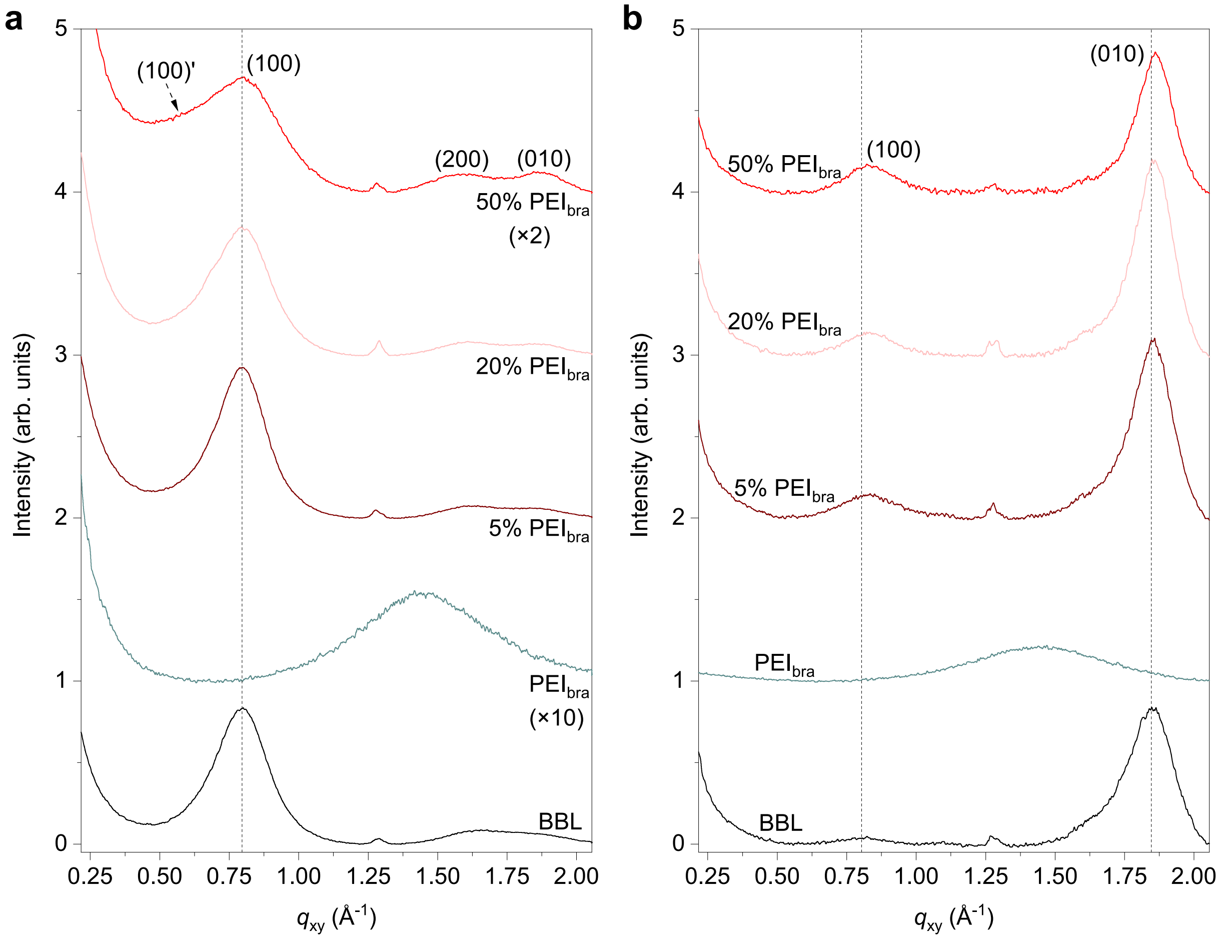

Supplementary Figure 3 | GIWAXS line-cuts analysis. a-b Out-of-plane (**a**) and in-plane (**b**) GIWAXS line-cuts of BBL, PEI_{bra}, and BBL:PEI_{bra} films with different PEI content. A clear shift of the (100) and (010) peaks are observed towards smaller molecular packings.

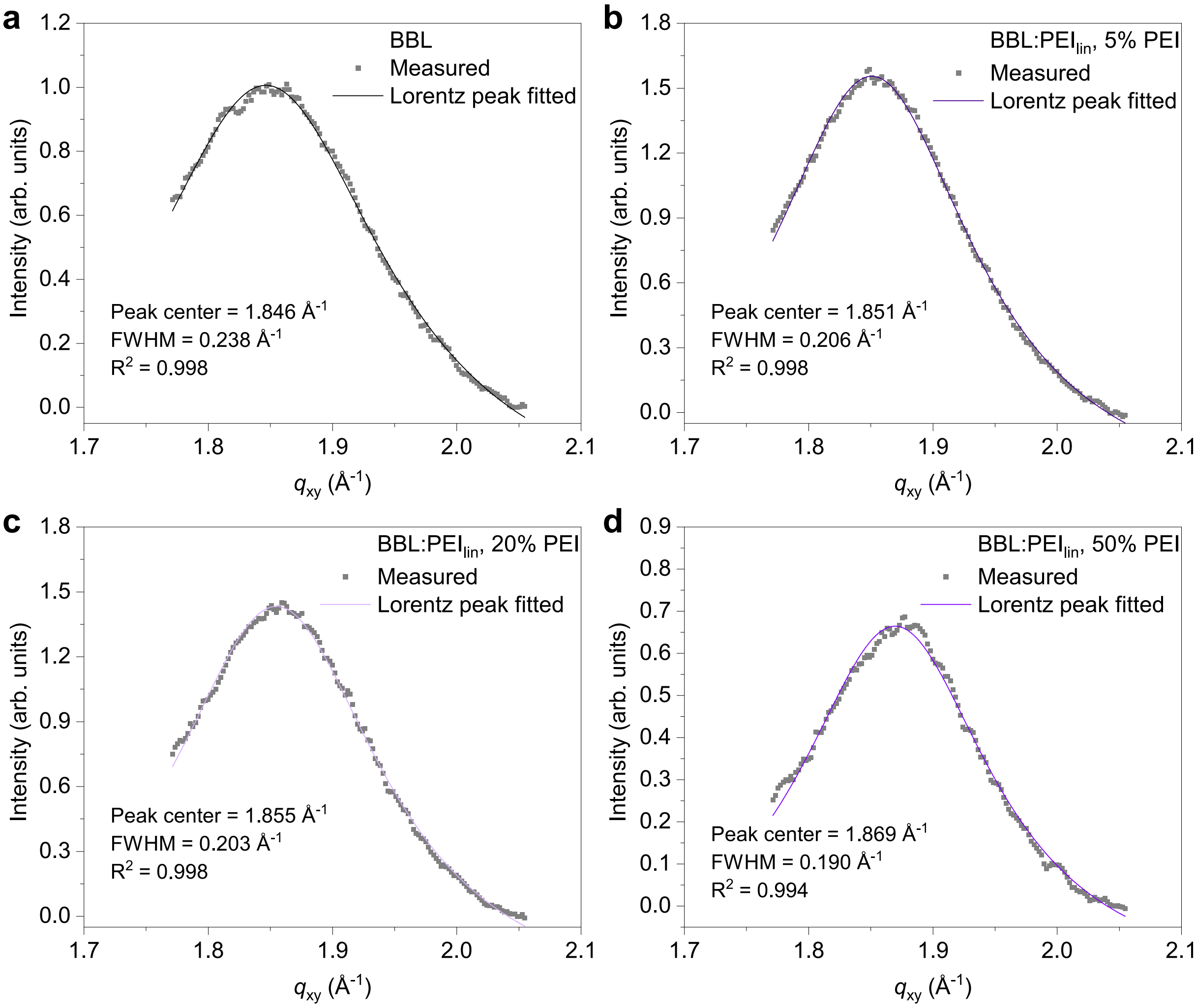

Supplementary Figure 4 | π-π stacking (010) diffraction analysis of BBL:PEI$_{lin}$. a-d Pristine BBL (**a**), and BBL:PEI$_{lin}$ with 5 wt-% (**b**), 20 wt-% (**c**), and 50 wt-% (**d**) PEI content.

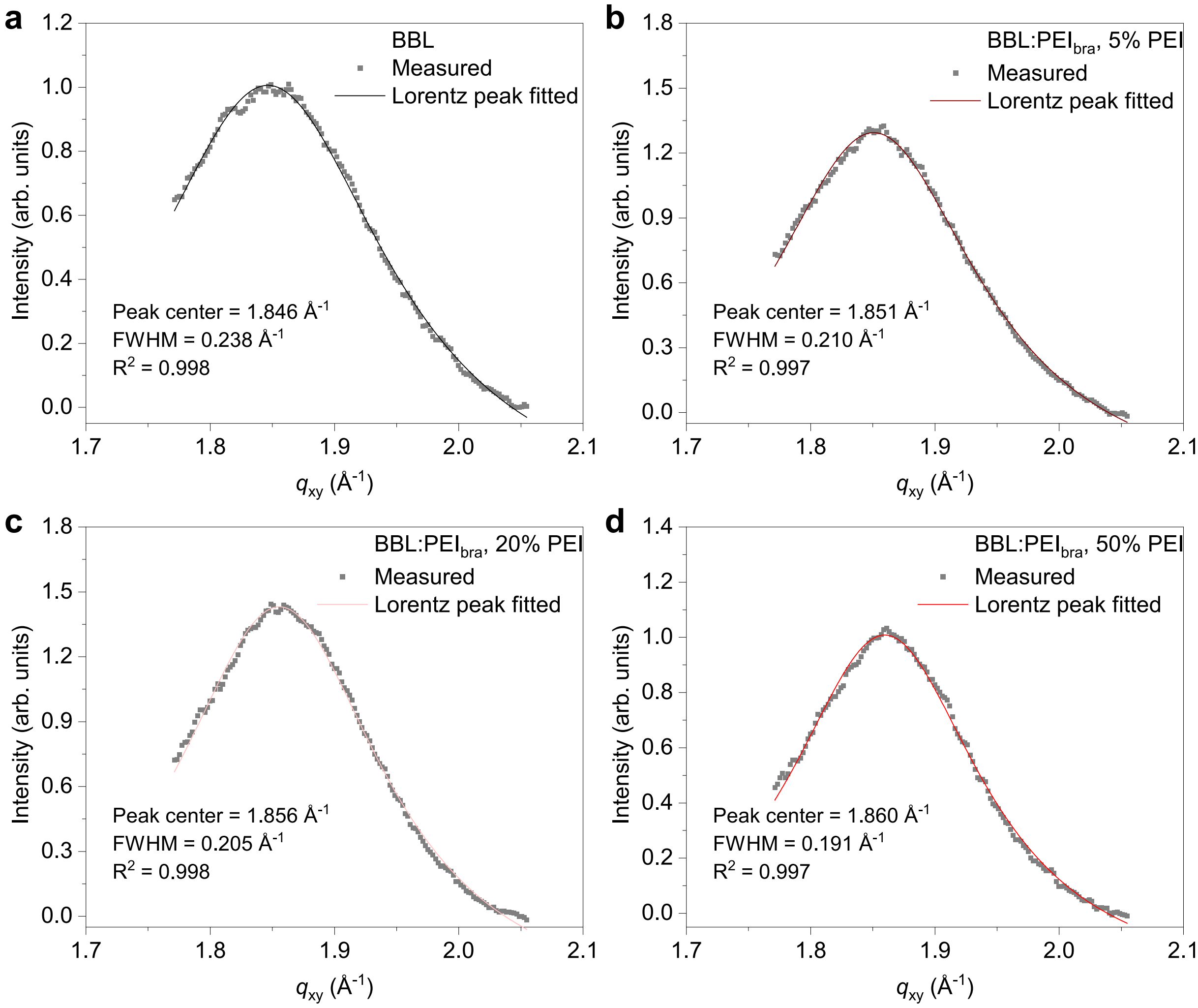

Supplementary Figure 5 | π-π stacking (010) diffraction analysis of BBL:PEI$_{bra}$. a-d Pristine BBL (**a**), and BBL:PEI$_{bra}$ with 5 wt-% (**b**), 20 wt-% (**c**), and 50 wt-% (**d**) PEI content.

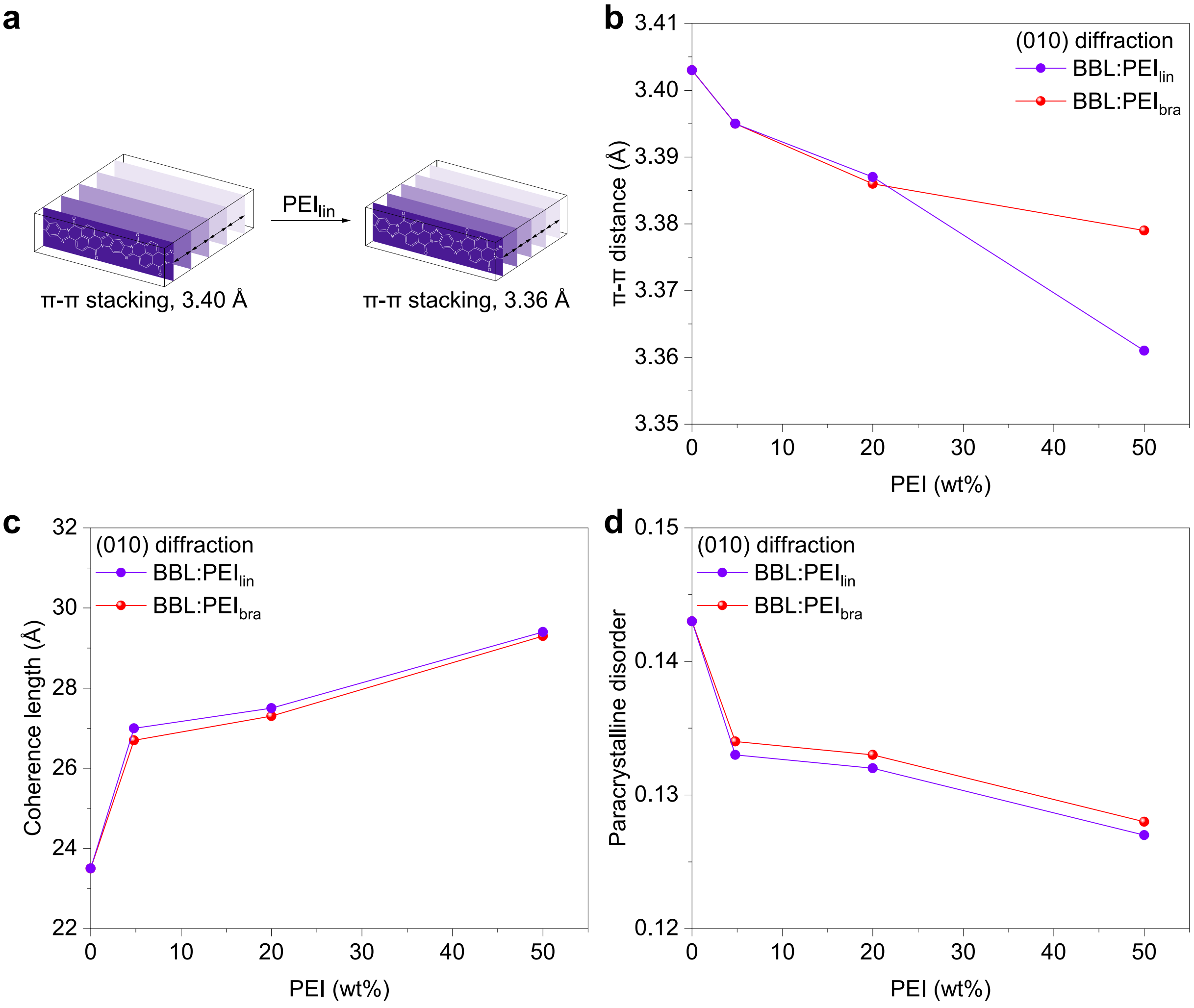

Supplementary Figure 6 | Summary of the (010) diffraction analysis. a Schematic of the effect of PEI on the BBL π-π stacking. **b-d** Evolution of the π-π stacking distance (**b**), coherence length (**c**), and paracrystalline disorder (**d**) of BBL when blended with various PEI$_{lin}$ or PEI$_{bra}$ content.

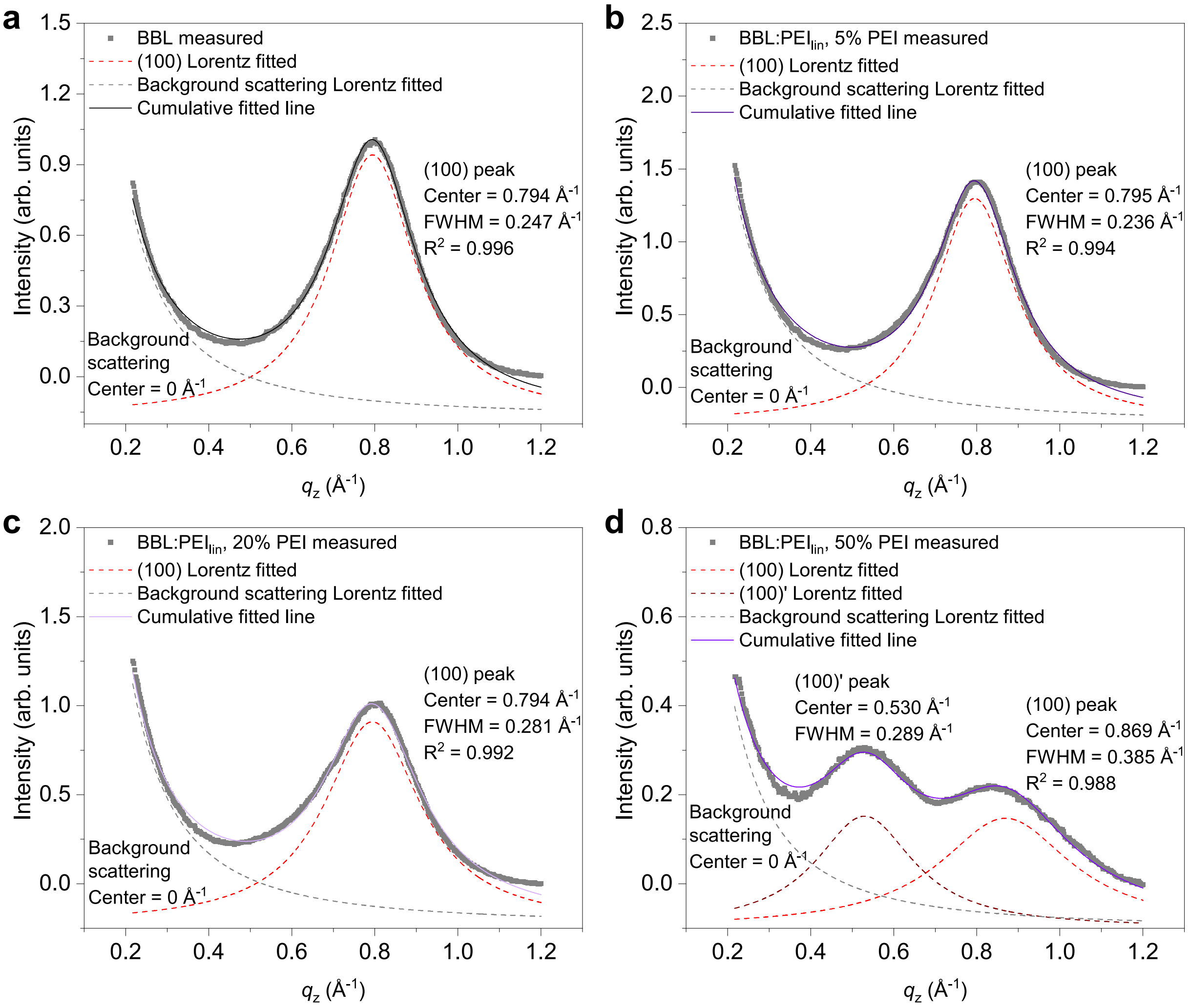

Supplementary Figure 7 | Lamellar (100) peak diffraction of BBL:PEI$_{lin}$. a-d Pristine BBL (**a**), and BBL:PEI$_{lin}$ with 5 wt-% (**b**), 20 wt-% (**c**), and 50 wt-% (**d**) PEI content. For BBL:PEI$_{lin}$ 50 wt-%, a new (100)' peak appears.

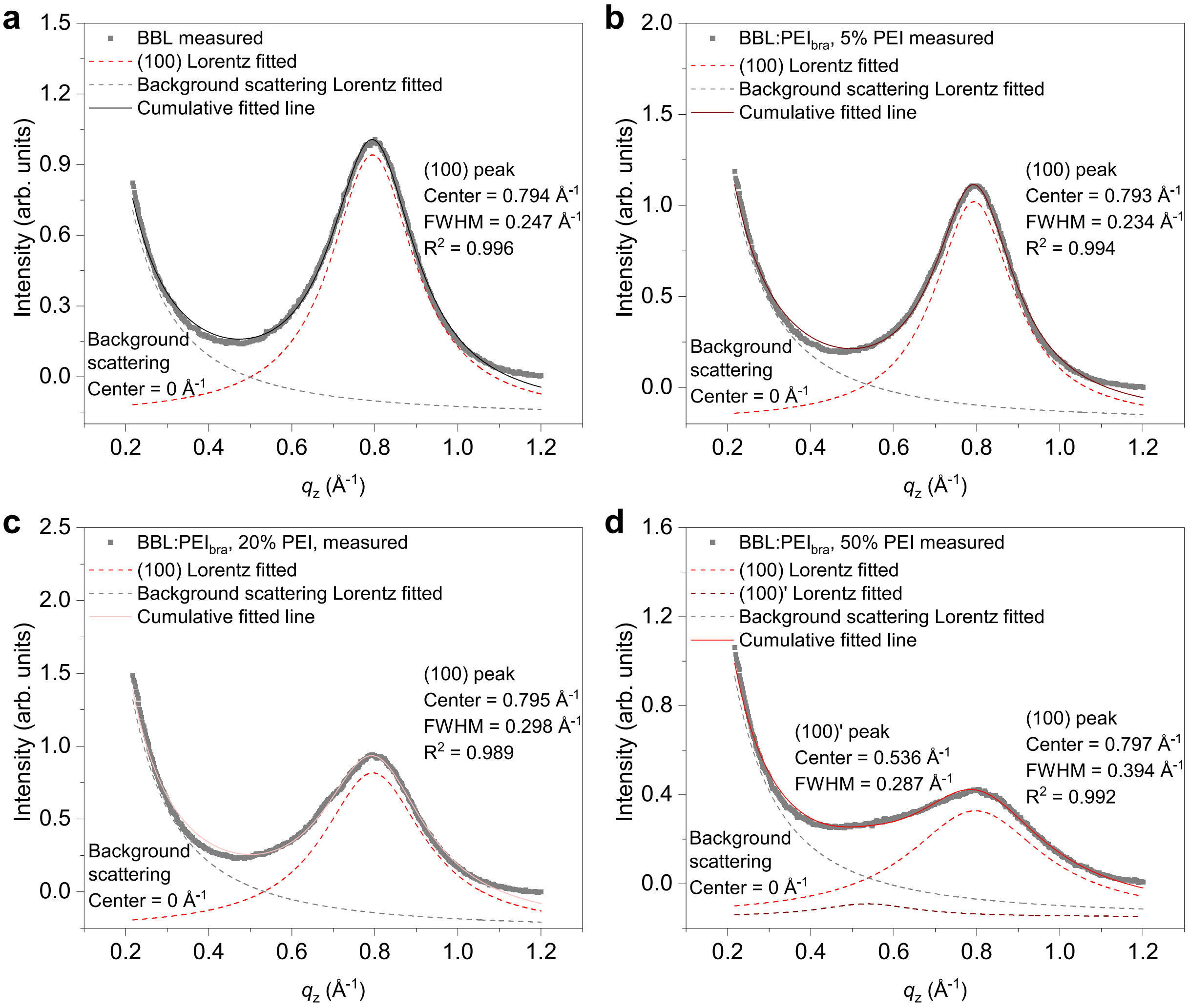

Supplementary Figure 8 | Lamellar (100) diffraction analysis of BBL:PEI$_{bra}$. a-d Pristine BBL (**a**), and BBL:PEI$_{bra}$ with 5 wt-% (**b**), 20 wt-% (**c**) and 50 wt-% (**d**) PEI content. For BBL:PEI$_{bra}$ 50 wt-%, the appearance of a shoulder is visible, attributed to the (100)' peak.

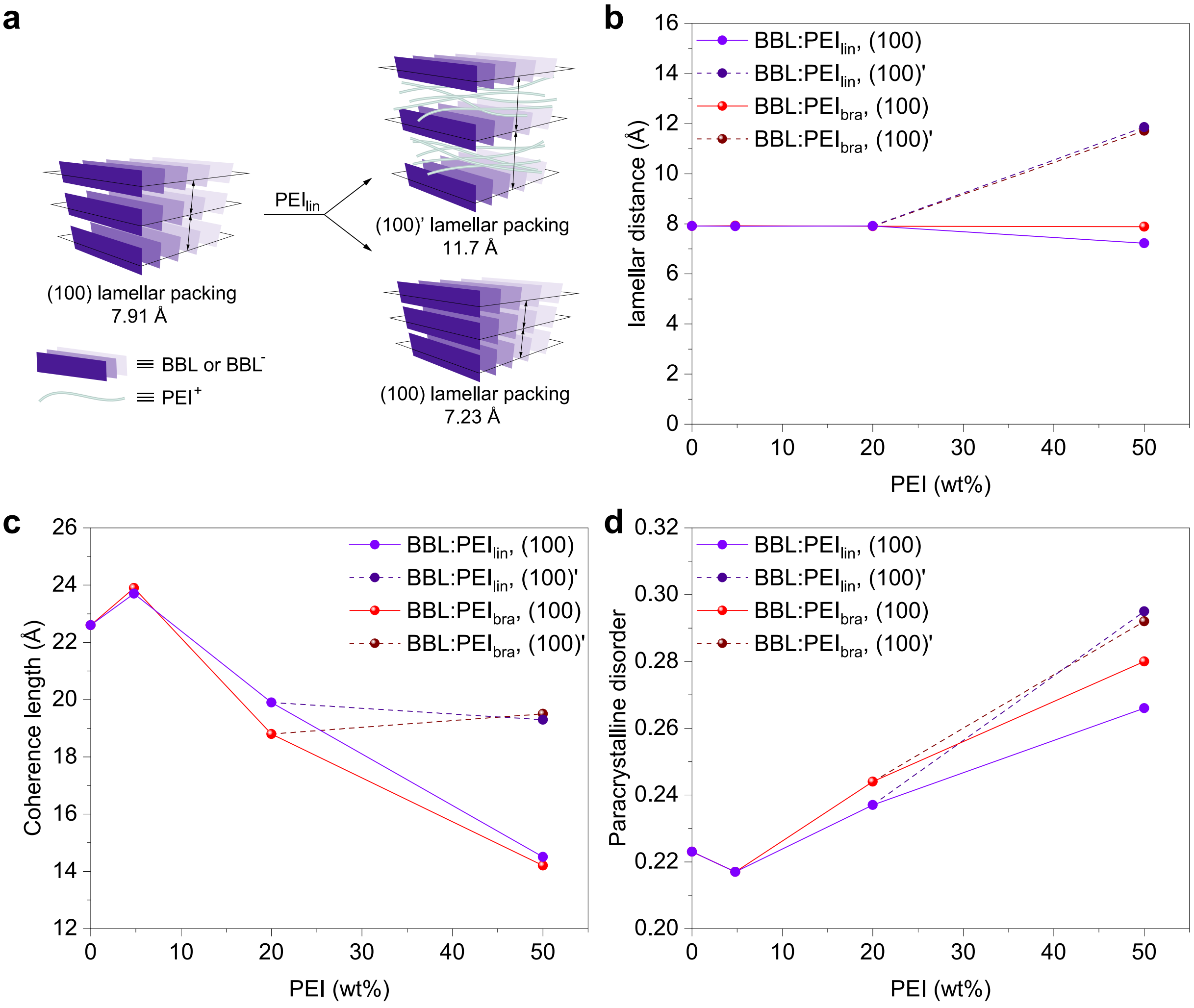

Supplementary Figure 9 | Summary of the (100) diffraction analysis. a Schematic of the effect of PEI on the BBL lamellar packing. **b-d** Evolution of the lamellar distance (**b**), coherence length (**c**), and paracrystalline disorder (**d**) of BBL when blended with various PEI_{lin} or PEI_{bra} content.

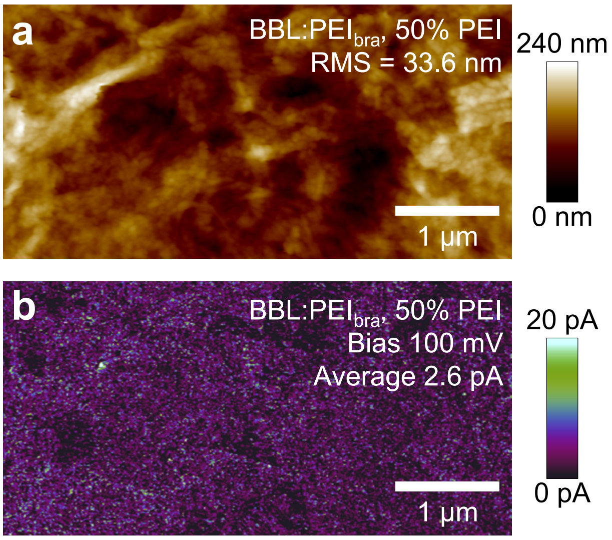

Supplementary Figure 10 | AFM and C-AFM analysis. a-b Atomic force microscope (AFM) analysis and corresponding conductive AFM (C-AFM) analysis of BBL:PEI$_{lin}$ (**a**, height image; **b**, current image).

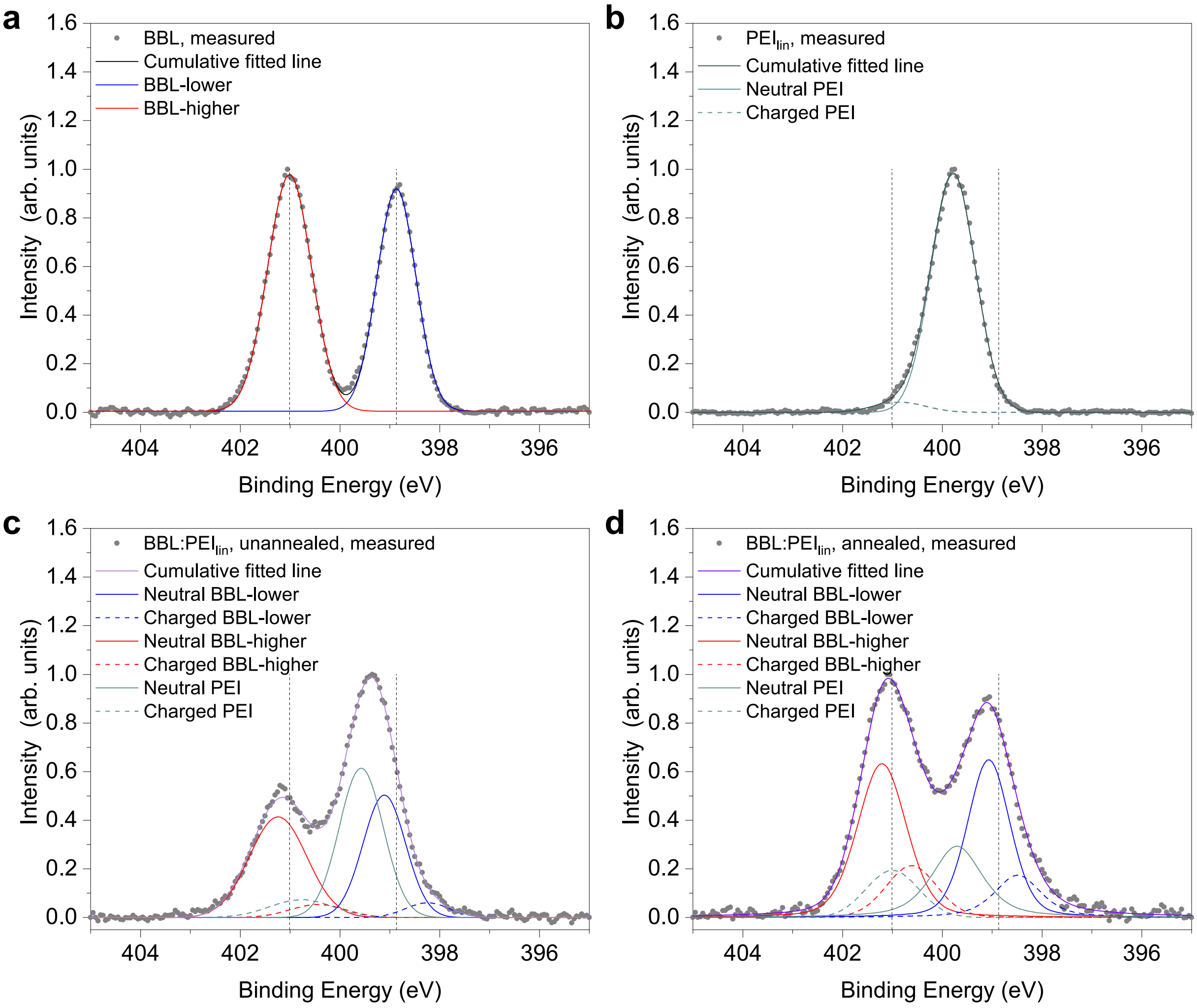

Supplementary Figure 11 | XPS analysis. a-d N(1*s*) XPS spectra of BBL (**a**), PEI$_{lin}$ (**b**), unannealed BBL:PEI$_{lin}$ (50 wt-% PEI, **c**), and annealed BBL:PEI$_{lin}$ (**d**). Pure BBL show two peaks at 389.9 eV and 401.0 eV relative to the two different nitrogen sites in BBL, whereas pure PEI$_{lin}$ has a main peak at 399.8 eV. For not annealed BBL:PEI$_{lin}$ blend, the low binding energy peak consists of partially overlapping peaks from neutral BBL and PEI$_{lin}$, with an additional small component from negatively charged BBL. The high binding energy peak is dominated by the neutral BBL with additional small components originating from charged BBL and PEI$_{lin}$. After annealing, a shift in the binding energy and a change in the relative intensity of the two peaks occur, as the charged BBL and PEI$_{lin}$ contributions increase whereas the neutral PEI$_{lin}$ and BBL decrease in relative intensity, suggesting significantly increased doping of the BBL chains.

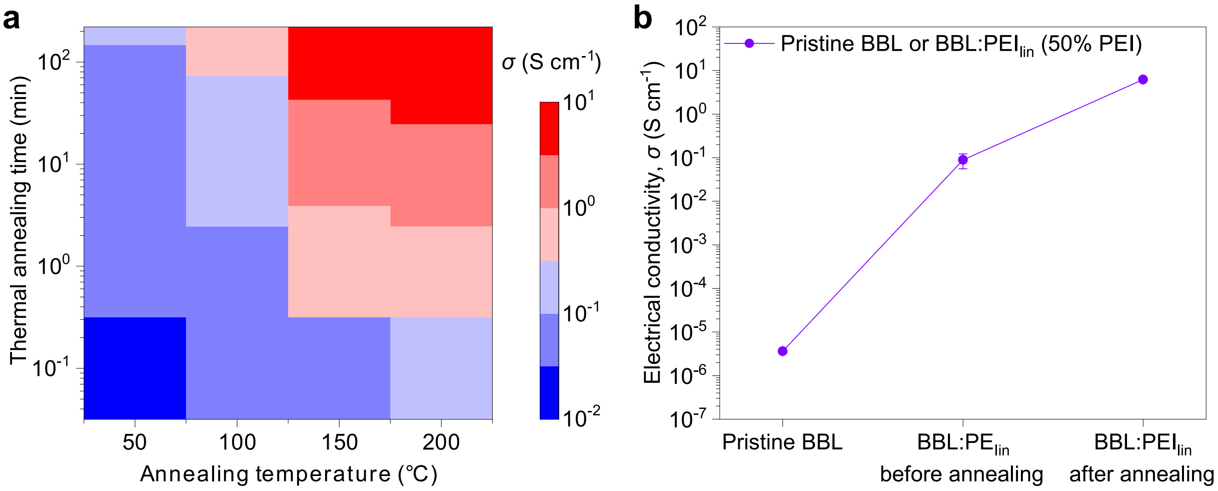

Supplementary Figure 12 | Effect of thermal treatment on the electrical conductivity. a Thermal annealing time vs annealing temperature for a BBL:PEI$_{lin}$ film (50 wt-% PEI content). While an electrical conductivity of 1 S cm^{-1} can be reached for low temperature or short annealing time (typically 100 °C for 10 min or 150 °C for 5 min), the maximum conductivity of almost 8 S cm^{-1} is achieved for longer annealing times (i.e., 150 °C for 120 min or 200 °C for 90 min). **b** Comparison of the conductivity of pristine BBL and BBL:PEI$_{lin}$ before and after annealing. Error bars indicate the SD of ten experimental replicates.

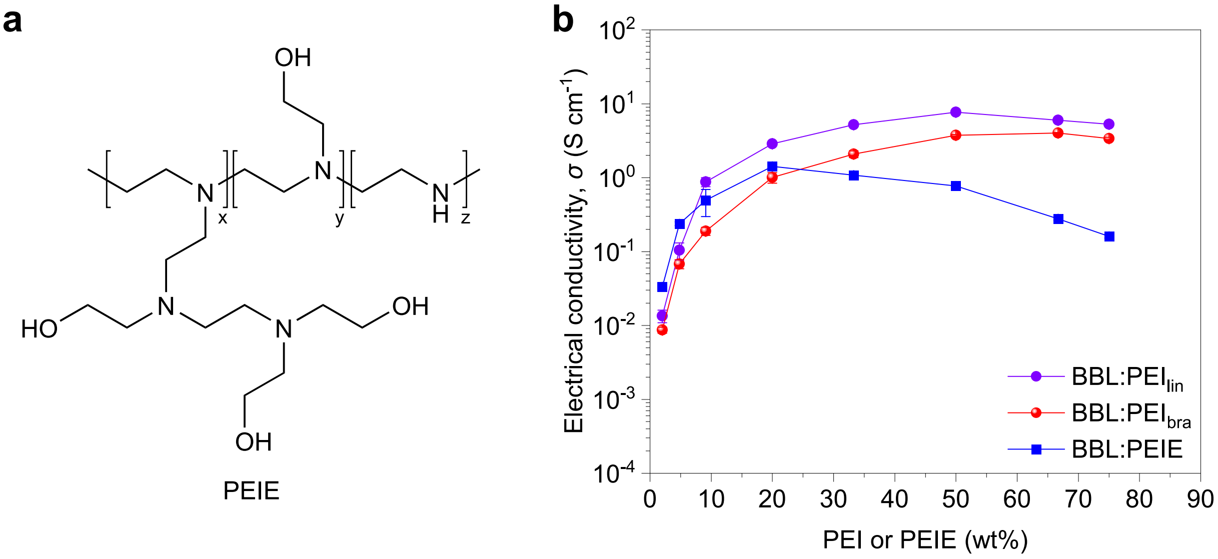

Supplementary Figure 13 | Electrical conductivity. a Chemical structure of PEIE (polyethylenimine ethoxylated). **b** Electrical conductivity of BBL:PEI$_{lin}$, BBL:PEI$_{bra}$ and BBL:PEIE films for different blend ratios. Error bars indicate the SD of ten experimental replicates. We attribute the decrease in conductivity at high PEIE content to the large concentration of hydroxyl (-OH) groups in the branched structure of PEIE, that could act as trapping sites for electrons[1,2].

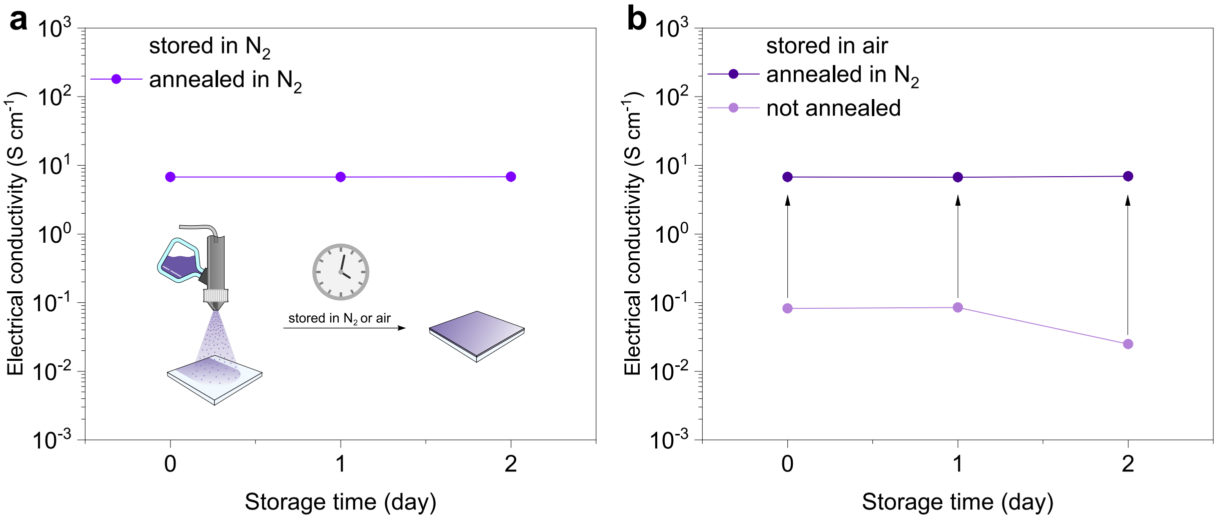

Supplementary Figure 14 | Pre-annealing stability test. a-b Electrical conductivity of BBL:PEI$_{lin}$ (50 wt-% PEI content) films stored in nitrogen atmosphere (**a**) or in air directly after the spray-coating (**b**) before thermal annealing. Error bars indicate the SD of ten experimental replicates. These results show that BBL:PEI can be stored either in air or under inert atmosphere prior to the thermal annealing. While the conductivity of pristine BBL:PEI$_{lin}$ is slightly affected by the ambient conditions, an equal conductivity level with samples stored in nitrogen is reached after annealing.

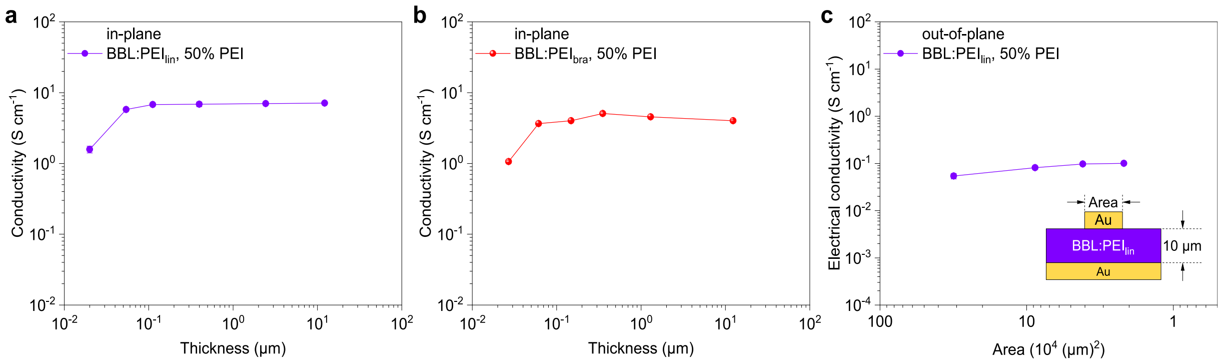

Supplementary Figure 15 | In-plane and out-of-plane conductivity. a-b Thickness-dependence of the in-plane conductivity of BBL:PEI$_{lin}$ (**a**) and BBL:PEI$_{bra}$ (**b**) with 50 wt-% PEI content. **c** Out-of-plane conductivity measured on a 10-μm-thick BBL:PEI$_{lin}$ film with different area gold electrodes. Error bars indicate the SD of ten experimental replicates. An average out-of-plane conductivity of 0.1 S cm^{-1} is measured, which is less than 2 orders of magnitude smaller than the in-plane conductivity for the same sample.

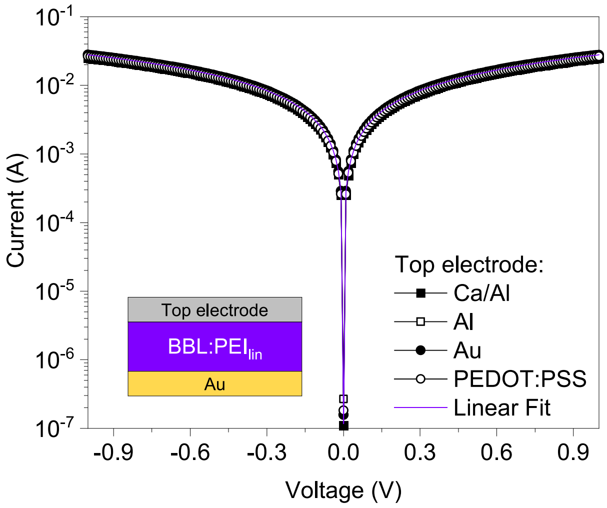

Supplementary Figure 16 | Ohmic contact measurements. Current-voltage characteristics of BBL:PEIlin (50 wt-% PEI content) films sandwiched between Au and various metal electrodes with work functions spanning from 5.1 eV (PEDOT:PSS) to 2.8 eV (Ca/Al). The BBL:PEIlin shows excellent ohmic contact with electrode having various work functions.

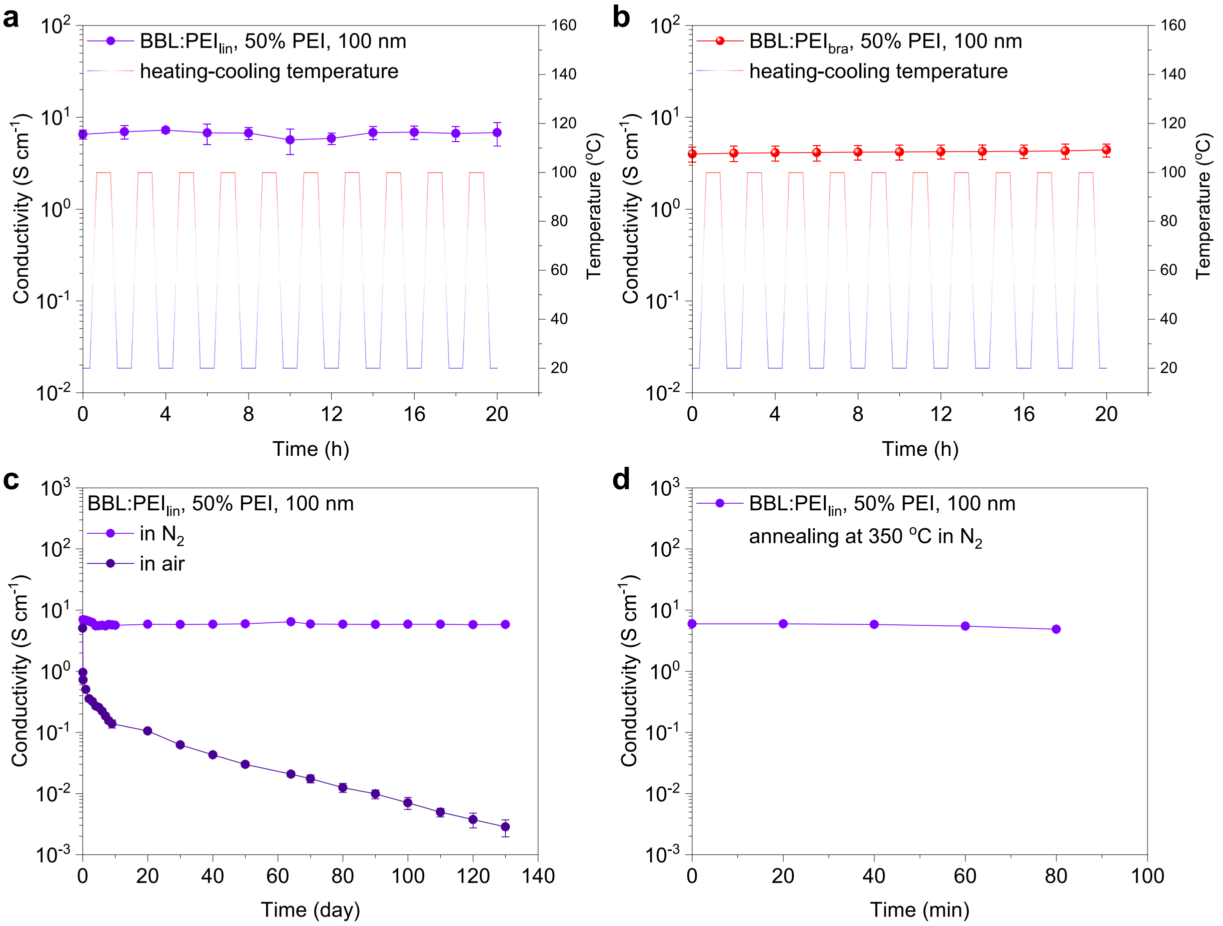

Supplementary Figure 17 | Stability over time. a-b Evolution of the conductivity of BBL:PEI_{lin} (**a**) and BBL:PEI_{bra} (**b**) with 50-wt% PEI content when subjected to thermal cycling between 20 °C and 100 °C, showing that the conductivity remains stable even after 10 cycles with no degradation for both blends. The temperatures were held for at least 10 min. **c** Evolution of the electrical conductivity over time for 100-nm-thick BBL:PEI_{lin} films stored in nitrogen or in air. While the samples stored in nitrogen show a very good stability over more than 100 days, the electrical conductivity of the thin samples stored in air drops to 0.1 S cm^{-1} after ten days. The use of encapsulation layers could extend the shelf stability of these thin films[3]. **d** Evolution of the electrical conductivity of BBL:PEI_{lin} (50 wt-%) annealed at 350 °C in nitrogen. After 80 min the conductivity drops by less than 30 %. Error bars indicate the SD of ten experimental replicates.

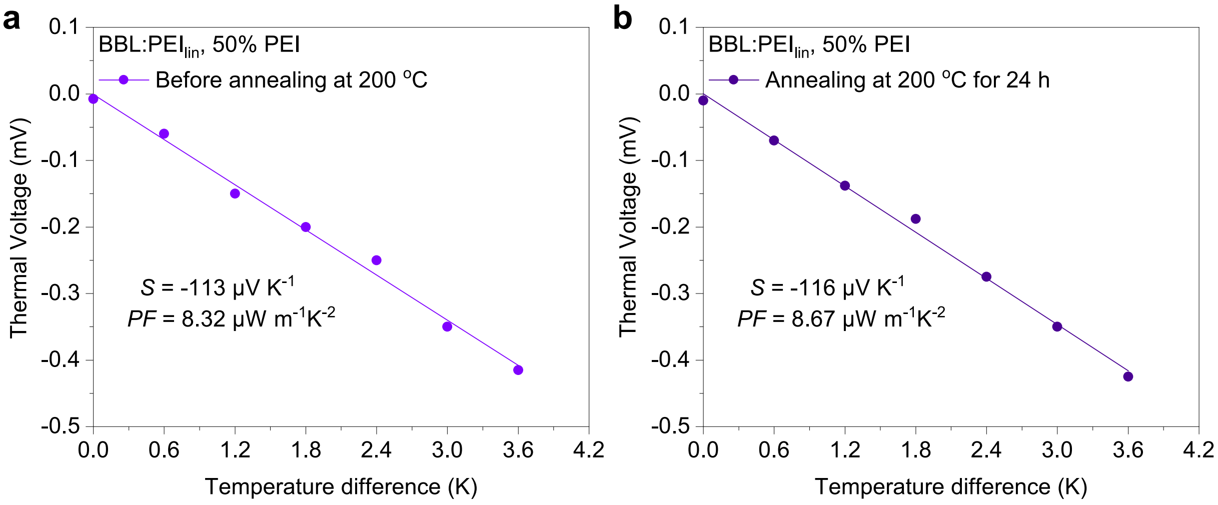

Supplementary Figure 18 | Thermal stability of the Seebeck coefficient. a-b Thermoelectric plots of BBL:PEI_{lin} films before (**a**) and after (**b**) annealing at 200 °C for 24 hours ($\sigma_{\text{before annealing}}$ = 6.53 S cm^{-1}; $\sigma_{\text{after annealing}}$ = 6.49 S cm^{-1}), demonstrating the exceptional thermal stability of the thermoelectric properties of this material.

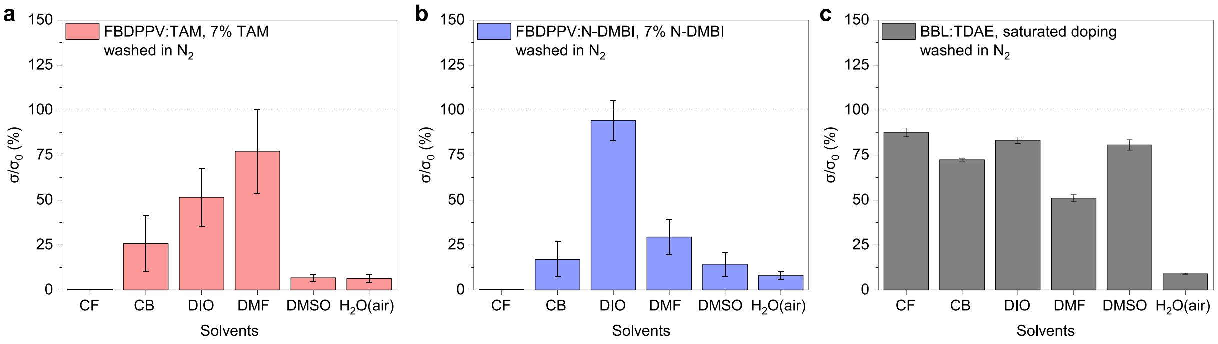

Supplementary Figure 19 | Solvent stability of other doped polymeric systems. a-b Stability tests for FBDPPV[4,5] polymer doped with triaminomethane (TAM, **a**) and 1,3-dimethyl-2-phenylbenzimidazoline (N-DMBI, **b**) when exposed to common organic solvents, showing a strong decrease in their electrical conductivity. **c** The same solvent test on BBL films doped with TDAE. While BBL:TDAE is more resistant to organic solvents than FBDPPV, it is still less stable than BBL:PEI. Error bars indicate the SD of ten experimental replicates.

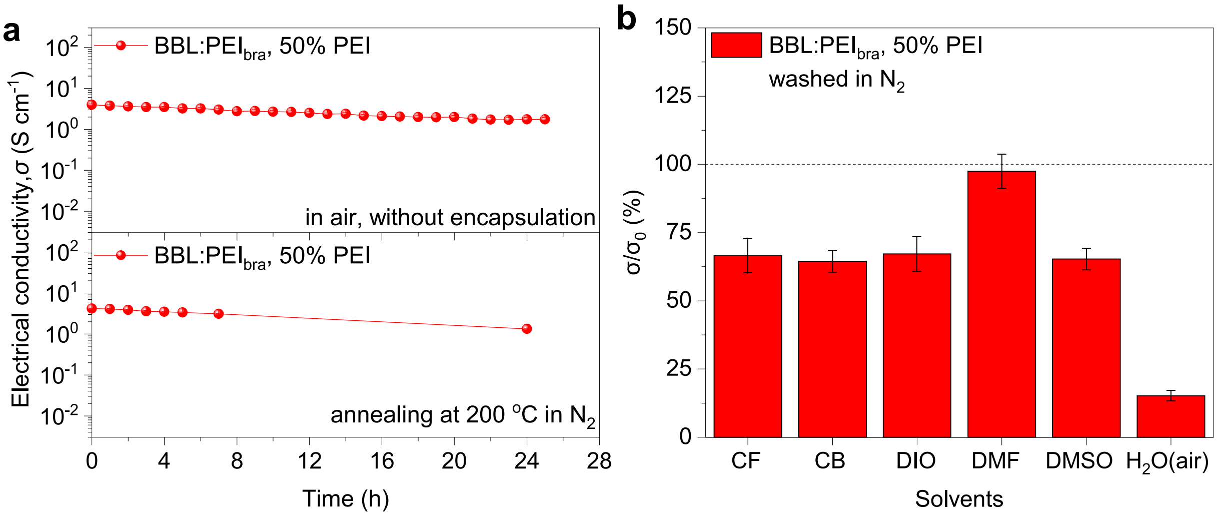

Supplementary Figure 20 | BBL:PEI$_{bra}$ stability. a Air and thermal (200 °C in N_2) stability of 10 μm thick BBL:PEI$_{bra}$ films evaluated by measuring electrical conductivity over 24 hours. **b** Stability of BBL:PEI$_{bra}$ exposed to common organic solvents, showing changes in electrical conductivity. Error bars indicate the SD of ten experimental replicates.

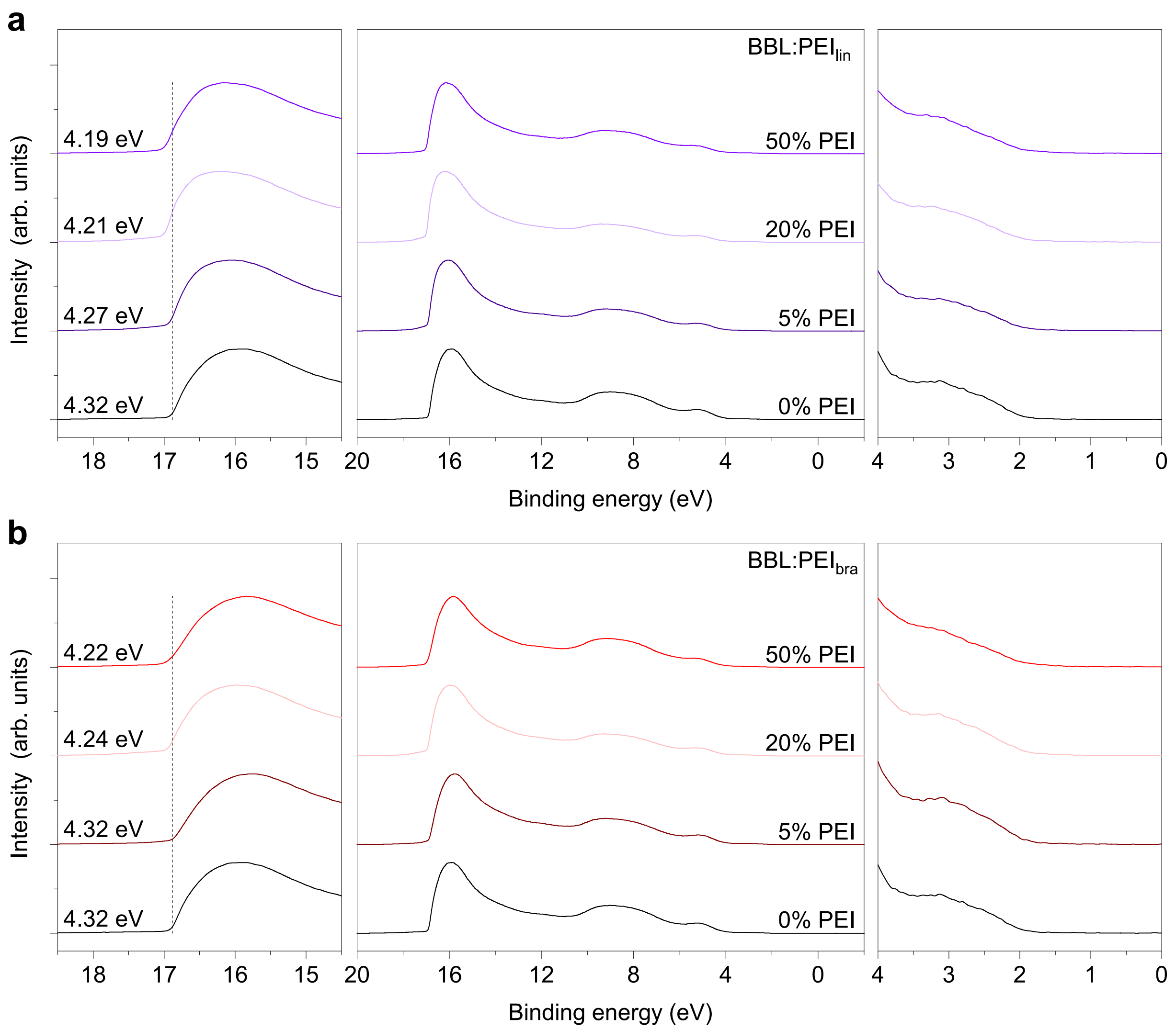

Supplementary Figure 21 | Work functions. a-b Ultraviolet photoelectron spectroscopy (UPS) analysis of BBL:PEI_{lin} (**a**) and BBL:PEI_{bra} (**b**) for different PEI content, recorded on gold substrates. The work-function shifts to lower values when compared to pristine BBL regardless of the type of PEI used.

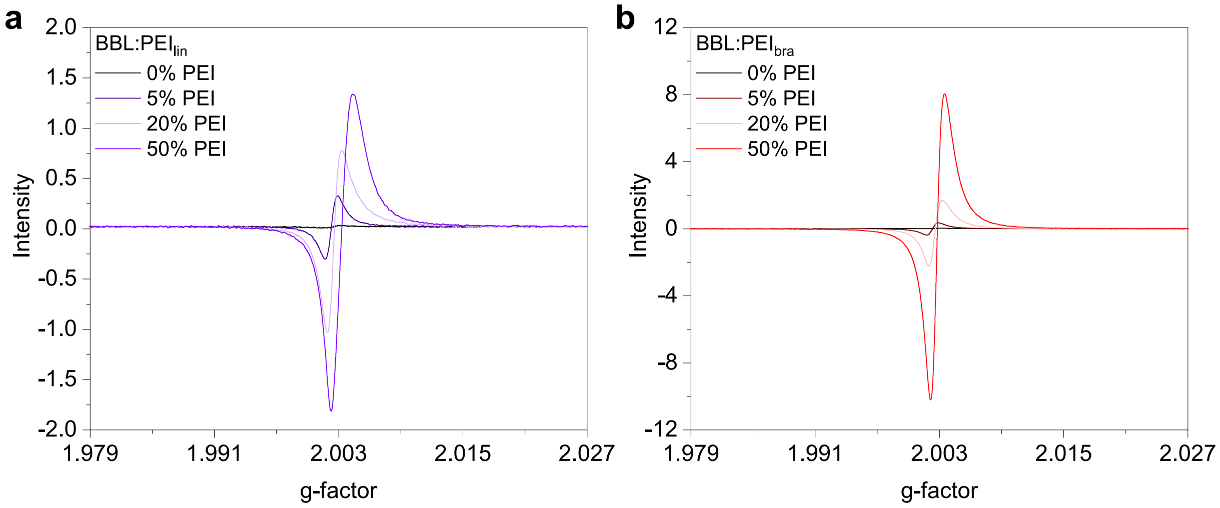

Supplementary Figure 22 | Spin density. a-b Electron paramagnetic resonance (EPR) spectra of BBL:PEI$_{lin}$ (**a**) and BBL:PEI$_{bra}$ (**b**), showing an increase in intensity upon increasing the PEI content. Note that the EPR intensity of BBL:PEI$_{bra}$ are stronger than for BBL:PEI$_{lin}$ with similar PEI content by weight. We attribute the asymmetry in the lineshape of the polaron EPR spectra to a *g*-factor anisotropy[6], which is commonly observed in conductive polymers[7,8].

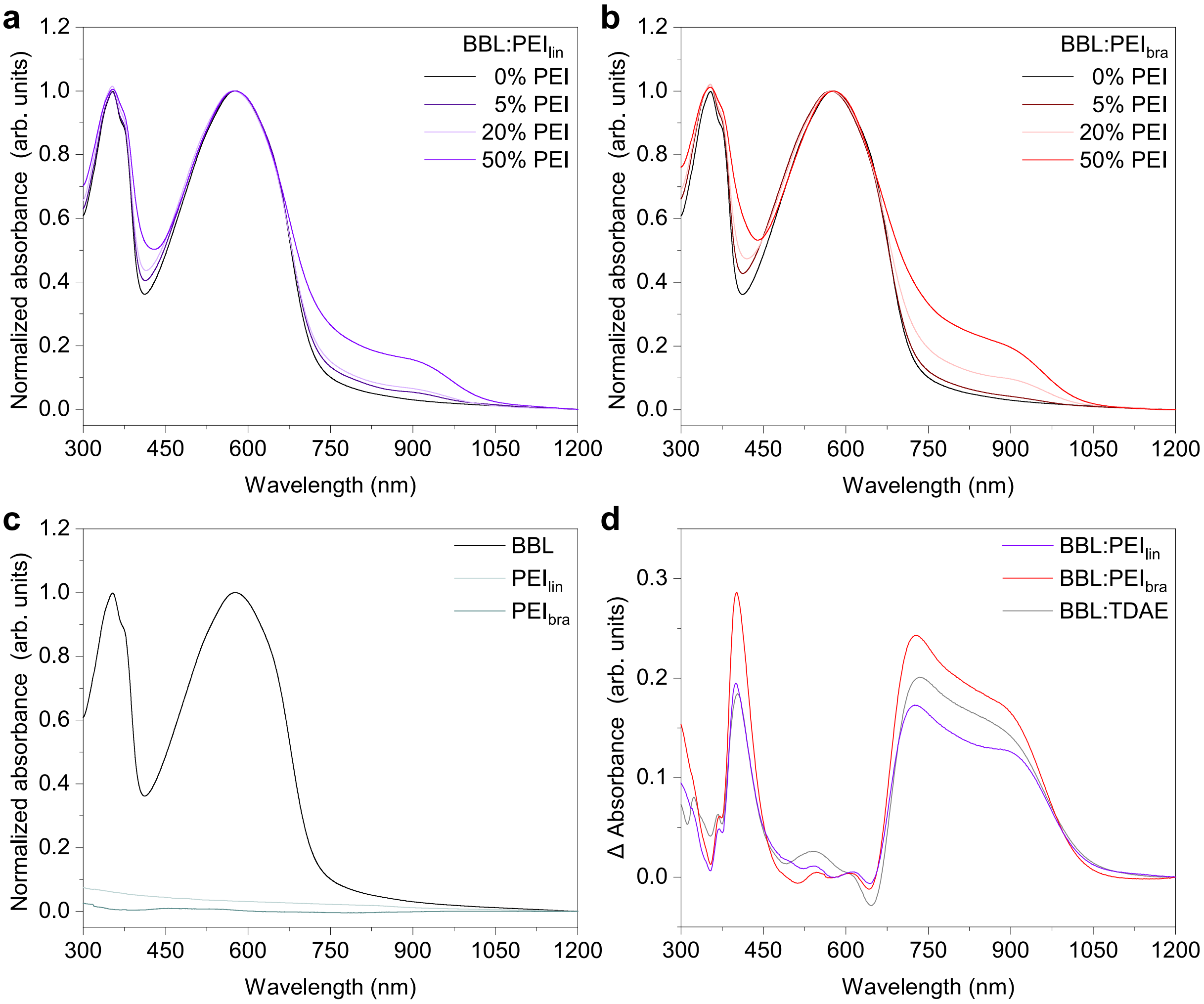

Supplementary Figure 23 | UV-Vis spectroscopy. a-c Normalized UV-Vis absorption spectra of BBL:PEI$_{lin}$ (**a**), BBL:PEI$_{bra}$ (**b**) and pristine BBL, PEI$_{lin}$, and PEI$_{bra}$ alone (PEI spectra are not normalized, **c**). The majority of the absorption is coming from BBL, while both PEI variants are almost transparent in the 300 nm to 1200 nm range. **d** Difference UV-Vis absorption spectra of 50 wt-% BBL:PEI$_{lin}$, BBL:PEI$_{bra}$ and BBL:TDAE calculated by subtracting the normalized pristine BBL absorbance, showing the appearance of polaronic peaks induced by the presence of PEI. Even though the polaronic absorption intensity is different due to different doping levels, the positions of the peaks are identical.

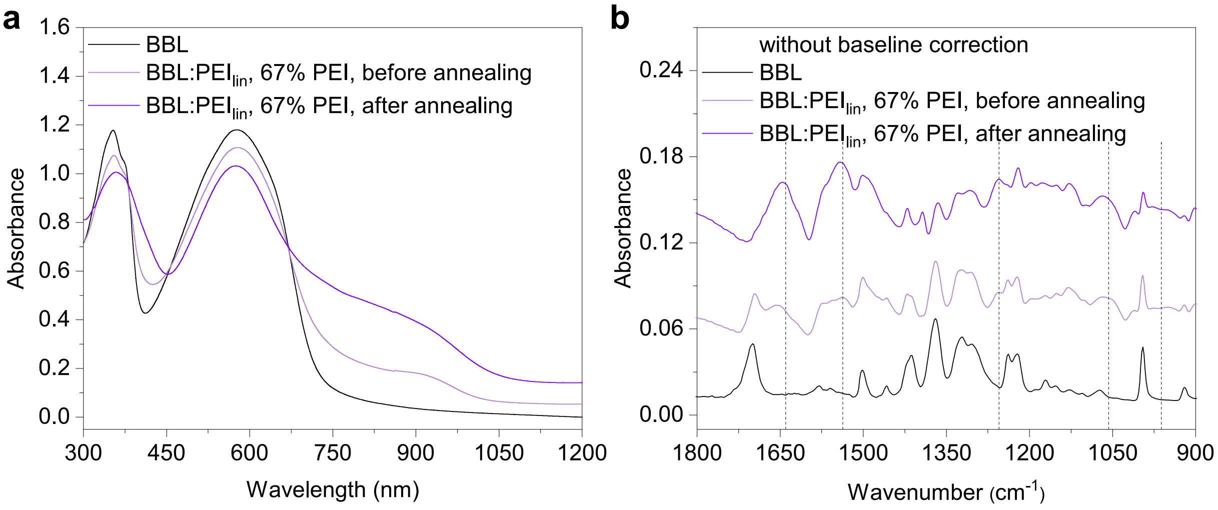

Supplementary Figure 24 | UV-Vis and FTIR absorbance of BBL:PEI$_{lin}$ before and after annealing. a Raw unnormalized UV-Vis absorption spectra illustrating how a polaronic absorption is visible already in the as-prepared BBL:PEI$_{lin}$ with 67 wt-% PEI before annealing. After annealing the same sample, the ground state absorption of BBL is decreased further, accompanied by an increase of the polaronic absorption. The pristine BBL absorption spectrum is a scaled spectrum for reference. **b** Raw unnormalized FTIR absorption spectra of the same sample before and after annealing. The strong polaronic IR absorptions at 1640 and 1535 cm^{-1} are clearly visible in the sample before annealing, and grow much more intense after annealing. For 67 wt-% PEI, the C=O vibration of pristine BBL at 1700 cm^{-1} disappears completely after annealing.

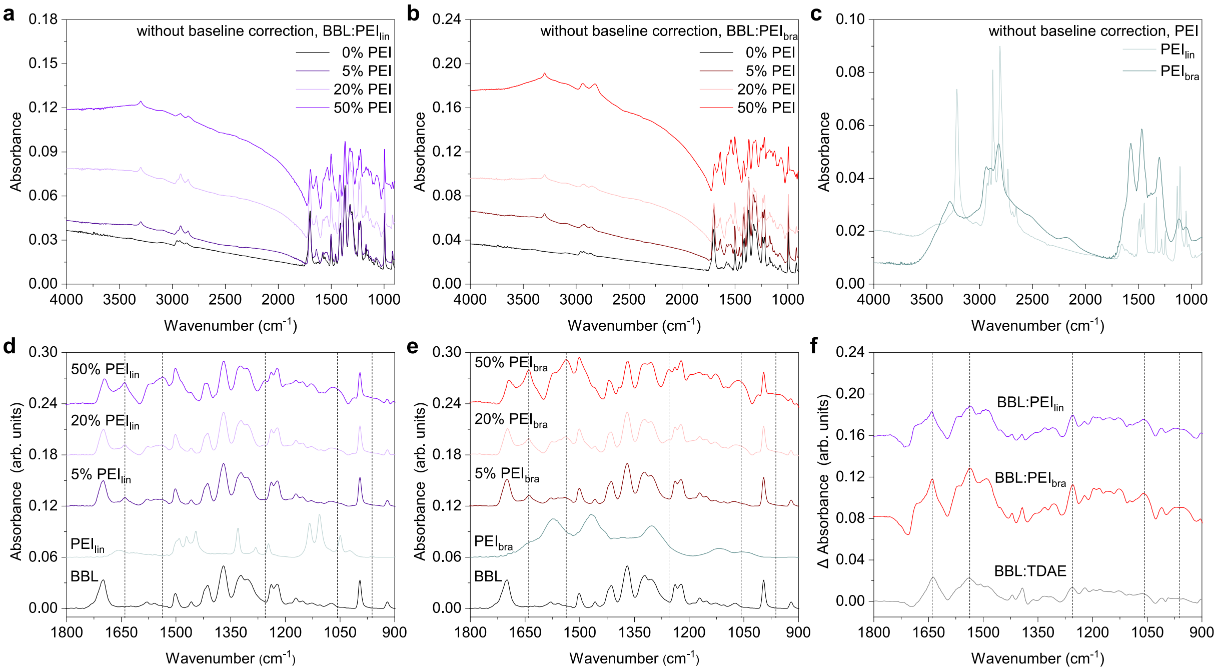

Supplementary Figure 25 | Fourier Transform Infrared spectroscopy (FTIR). a-c Raw unnormalized FTIR absorption spectra of BBL:PEI_{lin} (**a**) and BBL:PEI_{bra} (**b**) with different PEI content, and PEI_{lin} and PEI_{bra} alone (**c**). **d-e** Baseline corrected and normalized difference Fourier-transformed infrared (FTIR) absorption spectra of BBL:PEI_{lin} (**d**) and BBL:PEI_{bra} (**e**) for different PEI contents. The difference spectra were calculated by subtracting the baseline corrected absorption spectrum of BBL normalized at 1370 cm^{-1}, since the vibrations of PEI are relatively much weaker. The corresponding baseline corrected and scaled BBL and PEI spectra in **d** and **e** are shown for reference. **f** Comparison of the FTIR polaron absorption in 50 wt-% BBL:PEI_{lin}, BBL:PEI_{bra}, and BBL:TDAE.

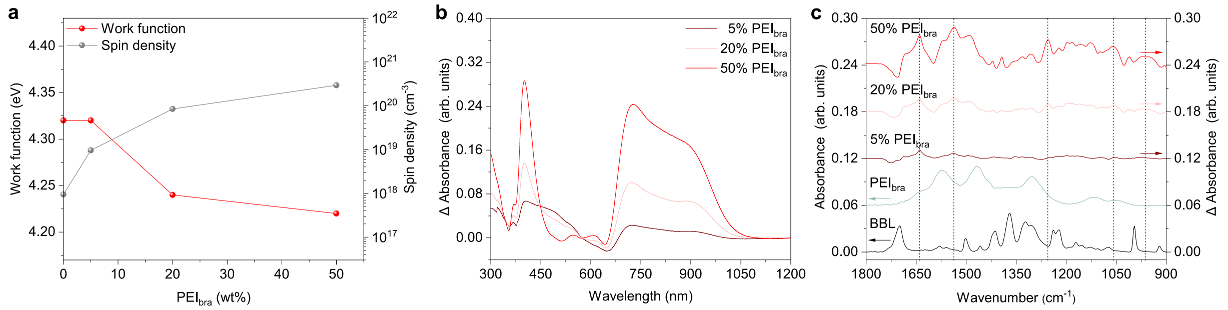

Supplementary Figure 26 | Spectroscopic confirmation of doping in BBL:PEI$_{bra}$ films. a Evolution of the work function measured by UPS and the spin density measured by EPR for BBL:PEI$_{bra}$ films with different PEI content. **b** Difference UV-Vis absorption spectra of BBL:PEI$_{bra}$ (50 wt-%) showing the evolution of polaron absorption induced by the increase in PEI content. **c** The corresponding difference FTIR absorption spectra of the same samples. The difference absorption spectra in (**b**) and (**c**) were obtained by subtracting the BBL spectrum from the BBL:PEI$_{bra}$ spectra after normalization at 575 nm and 1370 cm^{-1} for UV-Vis and FTIR, respectively.

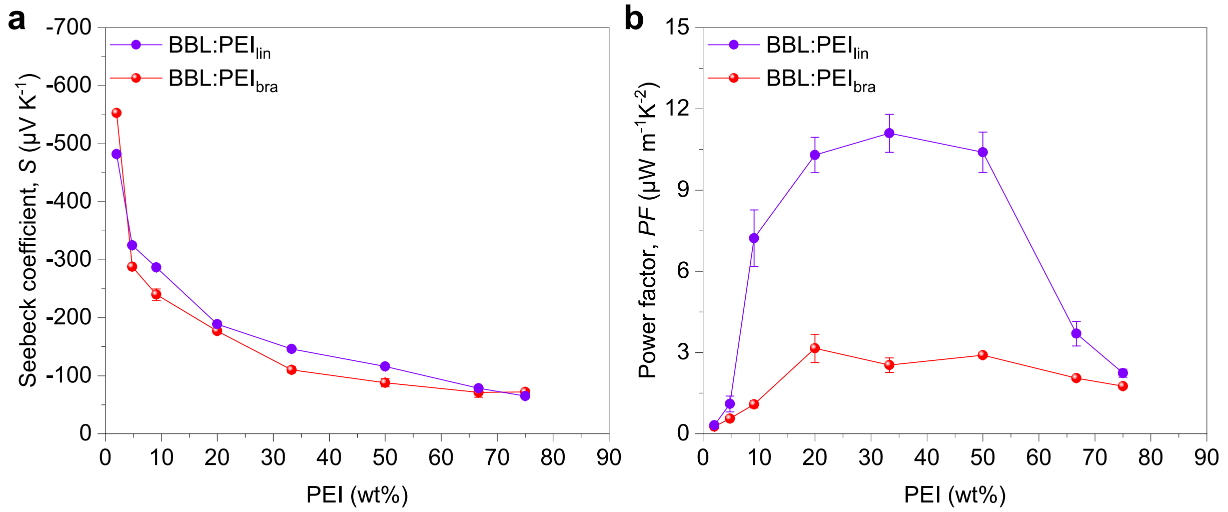

Supplementary Figure 27 | Thermoelectric performance. a-b Seebeck coefficients (**a**) and power factor (**b**) of BBL:PEI$_{lin}$ and BBL:PEI$_{bra}$. While both Seebeck coefficient curves are similar, a maximum power factor of 12 μW m^{-1} K^{-2} is obtained with BBL:PEI$_{lin}$ due to its higher conductivity compared to BBL:PEI$_{bra}$. Error bars indicate the SD of ten experimental replicates.

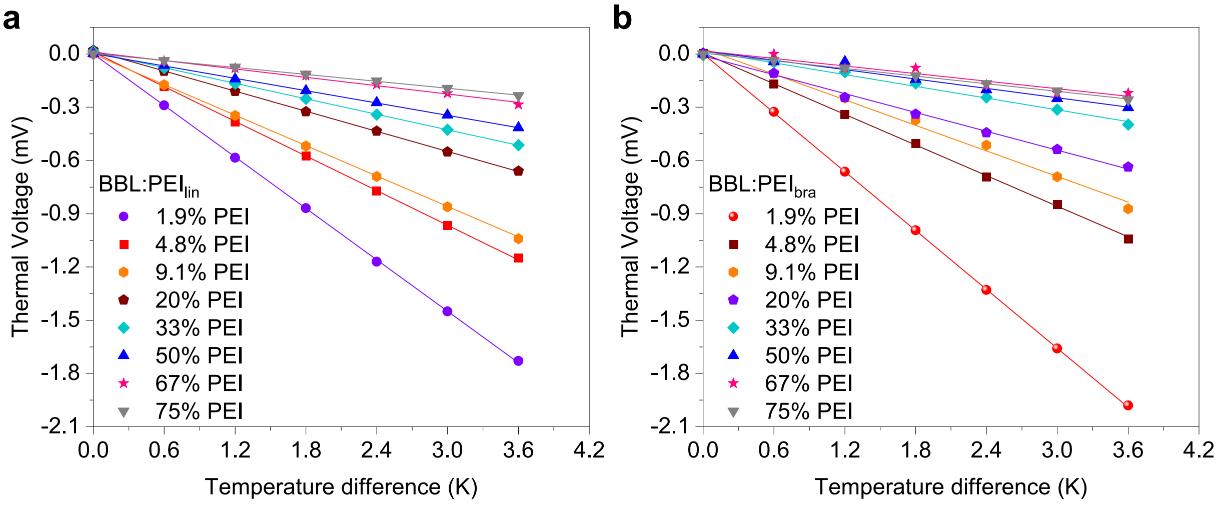

Supplementary Figure 28 | Thermoelectric performance. a-b Temperature difference dependent thermal voltage of BBL:PEI$_{lin}$ (**a**) and BBL:PEI$_{bra}$ (**b**).

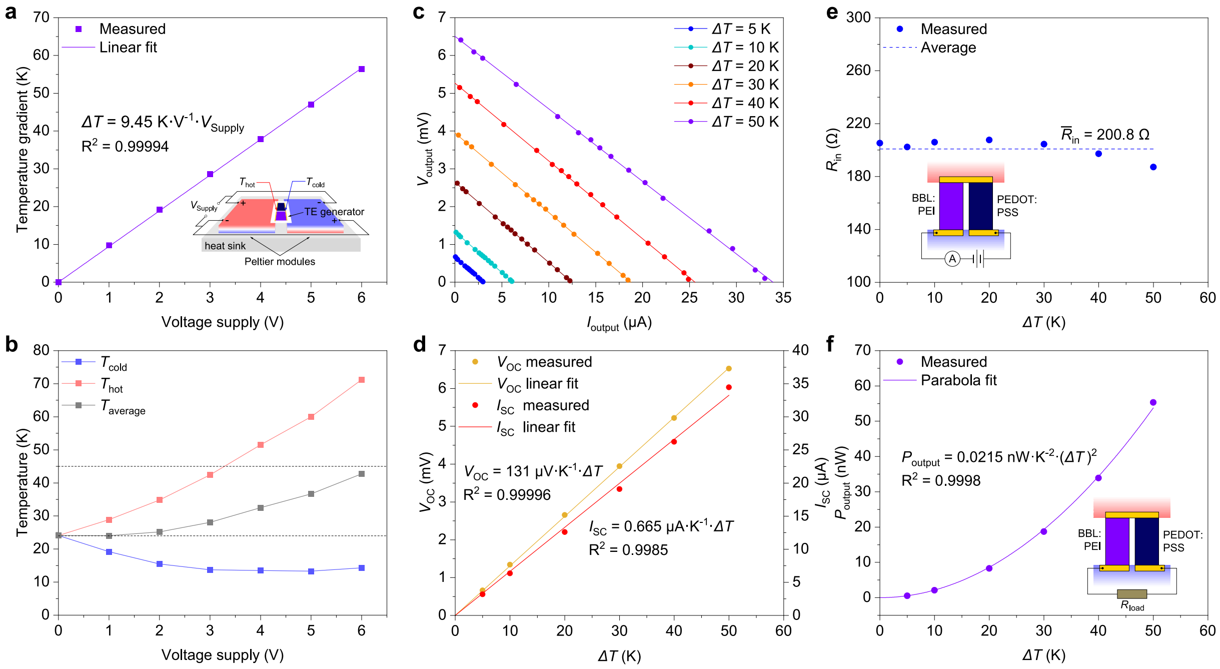

Supplementary Figure 29 | Thermoelectric generator characteristics. a-b Characterization of the double Peltier setup used for the thermoelectric module measurement with the calibration curve of the temperature gradient versus the voltage applied (**a**) and the corresponding absolute temperatures of the heating and cooling sides (**b**). **c** Voltage output (V_{output}) of the thermoelectric module as a function of the load current (I_{output}). **d-f** Open circuit voltage and short circuit current (**d**), internal resistance (**e**) and output power (**f**) of a planar thermoelectric module integrating one PEDOT:PSS *p*-leg and one BBL:PEI$_{lin}$ *n*-leg with gold contacts for different temperature gradients.

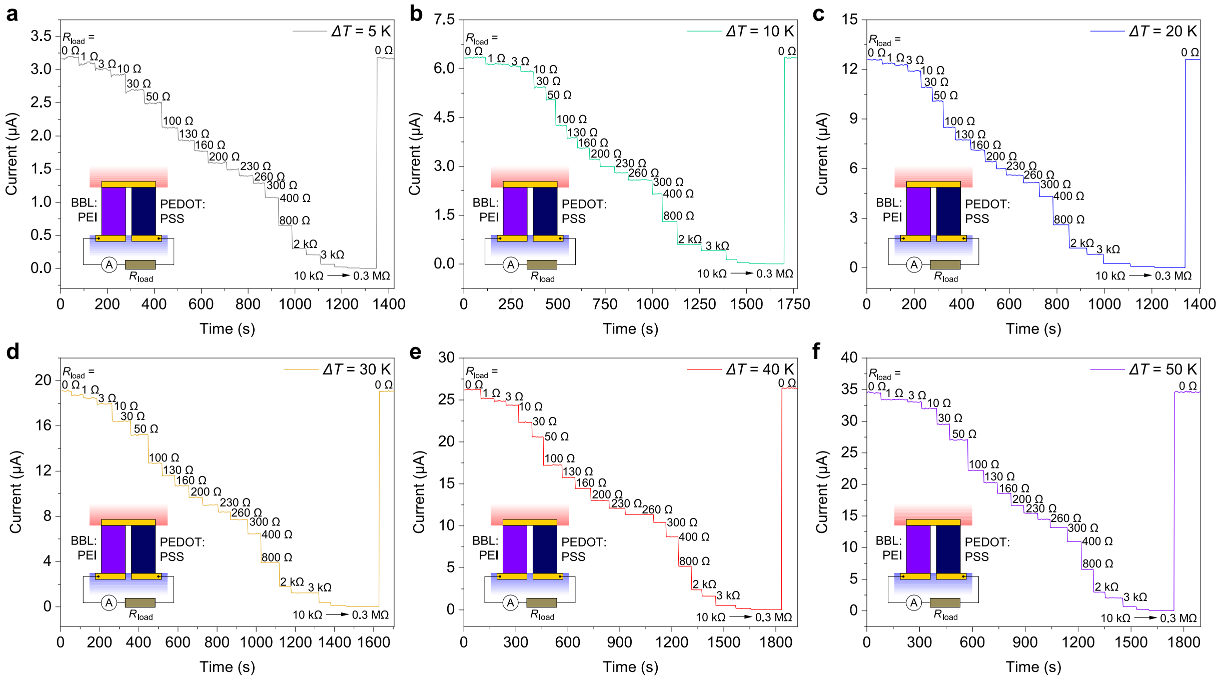

Supplementary Figure 30 | Thermoelectric generator characteristics. a-f Current of a planar thermoelectric module integrating one PEDOT:PSS *p*-leg and one BBL:PEI$_{lin}$ *n*-leg with gold contacts vs. time for different load resistances between 1 Ω and 0.3 MΩ recorded with thermal gradients of 5 K (**a**), 10 K (**b**), 20 K (**c**), 30 K (**d**), 40 K (**e**), and 50 K (**f**), showing a stable, immediately responding and fully reversible behaviour.

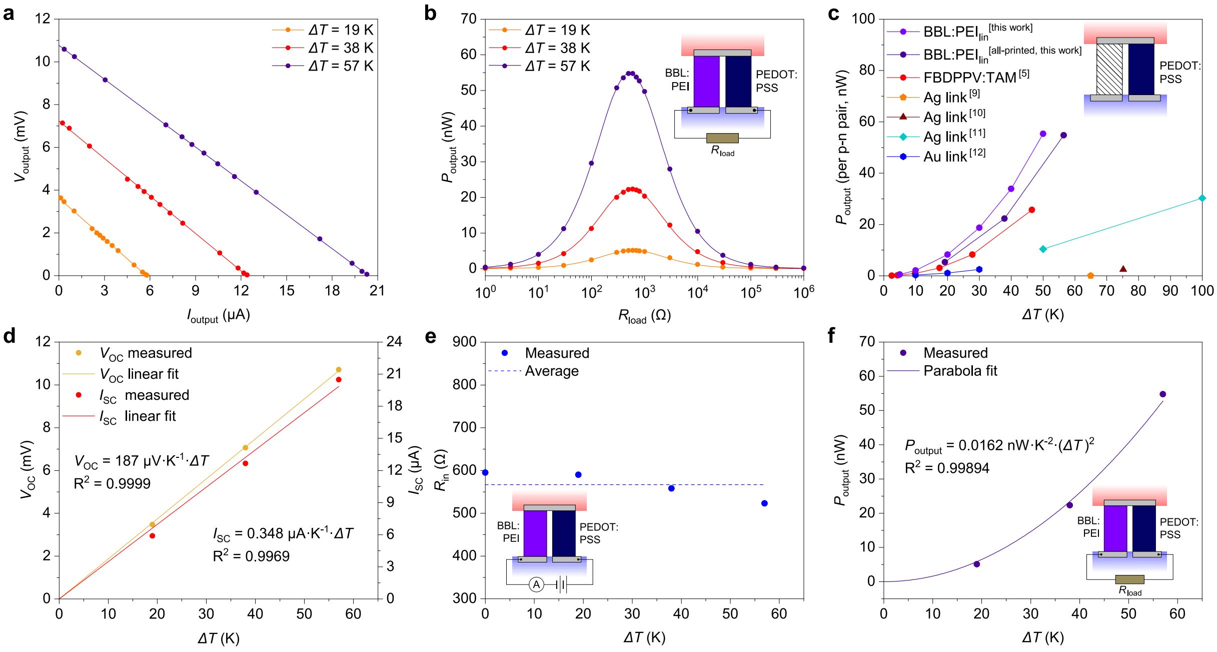

Supplementary Figure 31 | Thermoelectric generator characteristics. a Voltage output (V_{output}) of the thermoelectric module (integrating one PEDOT:PSS *p*-leg and one BBL:PEI$_{lin}$ *n*-leg as active material with painted silver contacts) as a function of the load current (I_{output}), recorded for different temperature gradients. **b** Output power versus load resistance of the thermoelectric module. **c** Summary of the output power recorded at different thermal gradients and comparison with thermoelectric modules featuring similar geometries in literature[5,9-12]. **d-f** Open circuit voltage and short circuit current (**d**), internal resistance (**e**), and output power versus different thermal gradients (**f**).

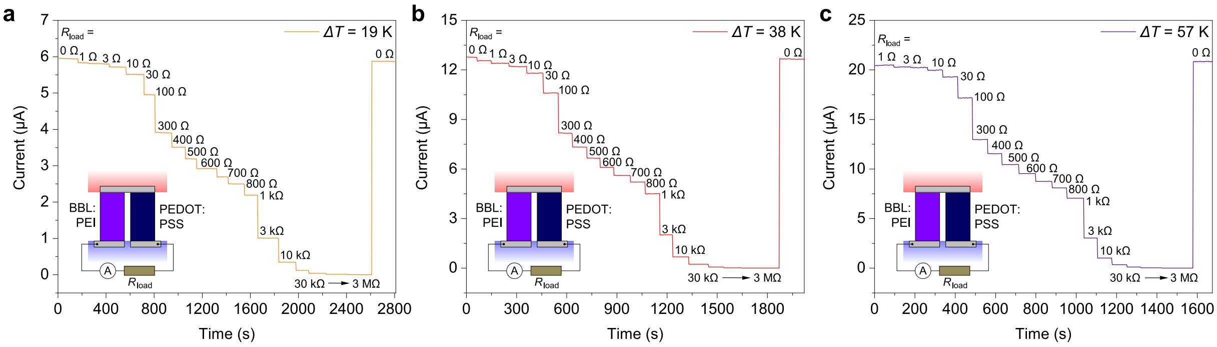

Supplementary Figure 32 | Thermoelectric generator characteristics. a-c Output current for different load resistances between 1 Ω and 3 MΩ recorded with a temperature gradient of 19.2 K (**a**), 37.9 K (**b**), and 56.5 K (**c**), showing a stable, immediately responding and fully reversible behaviour.

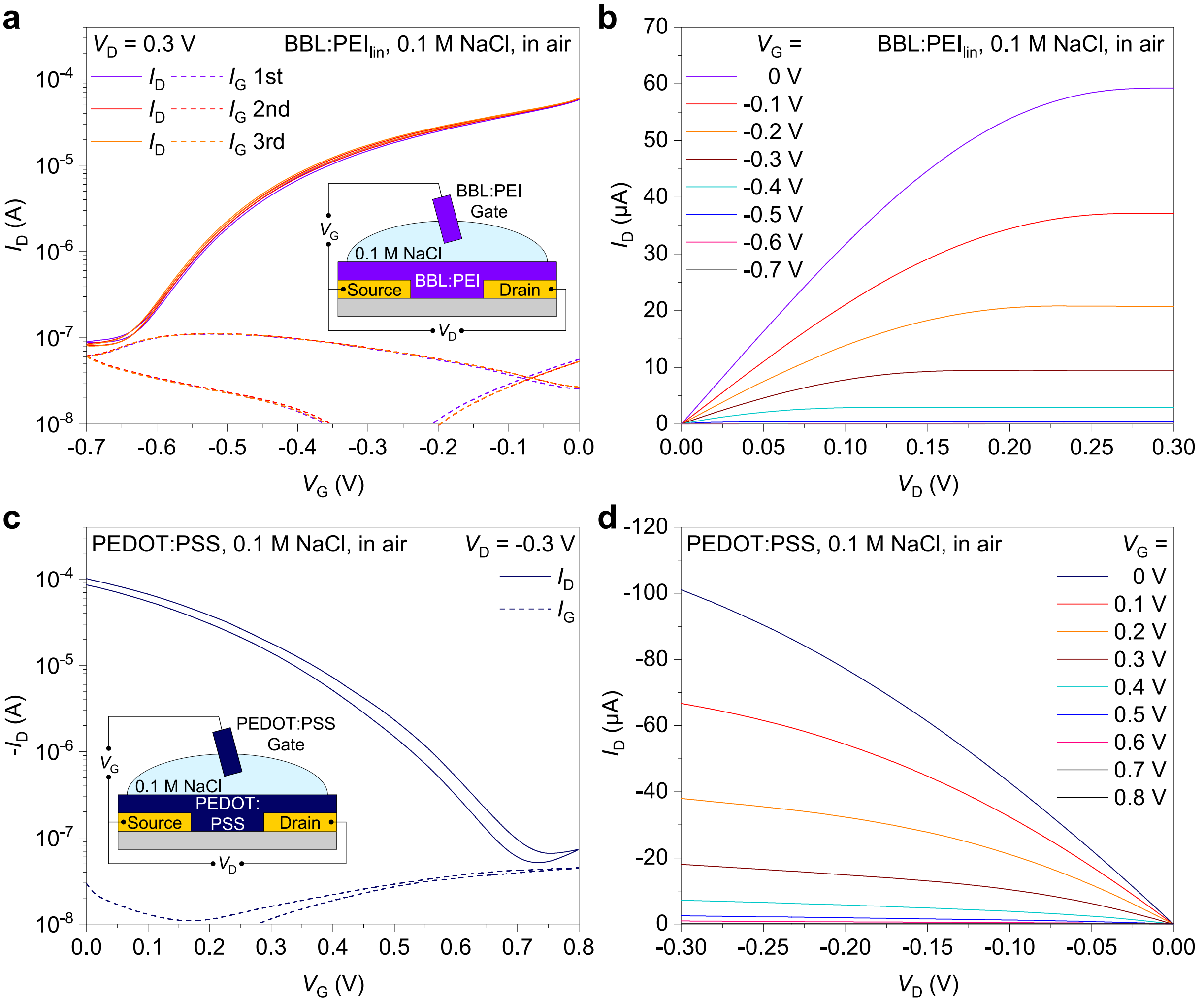

Supplementary Figure 33 | Depletion-mode organic electrochemical transistors. a-b Transfer curve (**a**) and output curve (**b**) of a BBL:PEI$_{lin}$-based organic electrochemical transistor (OECT) operating in air with 0.1 M NaCl aqueous electrolyte, showing a clear *n*-type conduction in the depletion-mode regime. **c-d** Transfer curve (**c**) and output curve (**d**) of a PEDOT:PSS-based OECT operating in air with 0.1 M NaCl aqueous electrolyte, showing a *p*-type depletion-mode behaviour.

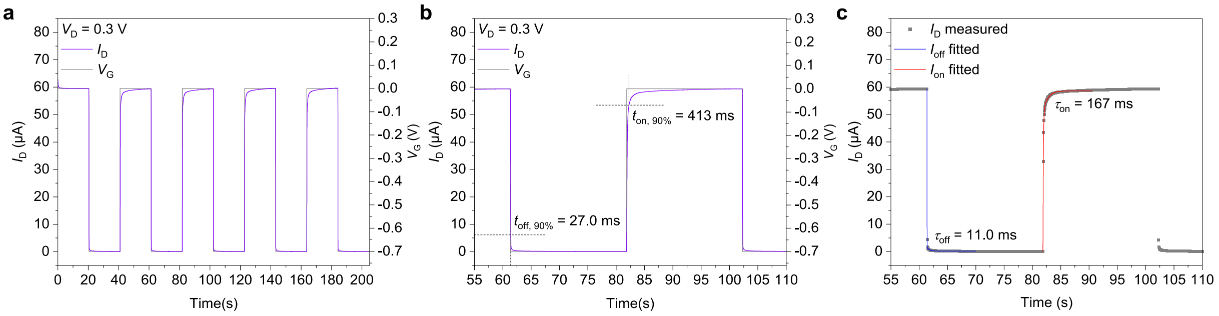

Supplementary Figure 34 | Temporal response of the n-type depletion mode OECTs. a Temporal response of BBL:PEI$_{lin}$ based OECTs to an applied square V_G. **b-c** Square gate voltage input displaying t_{on} and t_{off} for 90% of the signal of 27 ms and 413 ms, respectively (**b**), and exponential fits of the signal displaying t_{on} and t_{off} that are 11 and 167 ms, respectively (**c**).

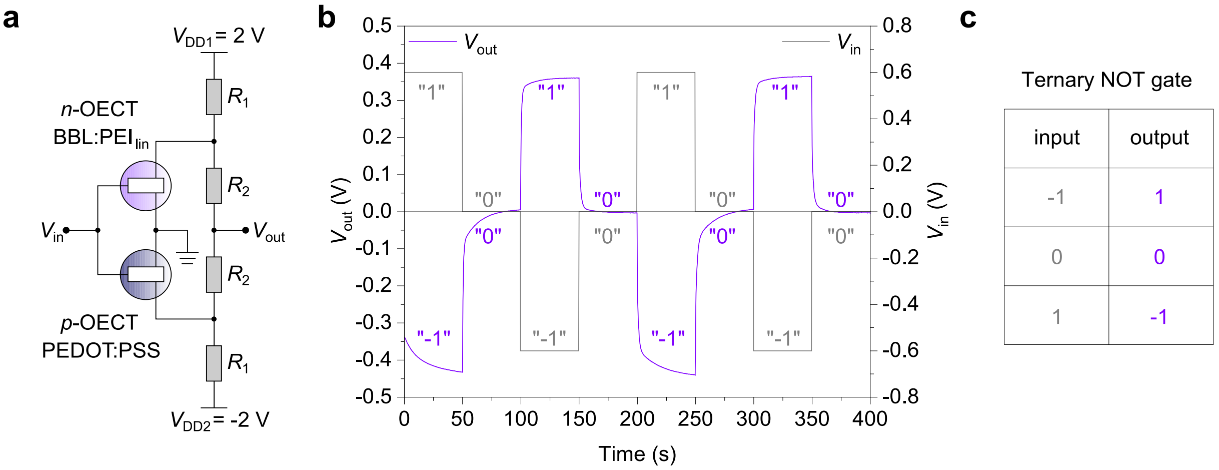

Supplementary Figure 35 | Ternary inverters. a Schematic of the ternary inverter integrating PEDOT:PSS and BBL:PEI$_{lin}$ as *p*-type and *n*-type depletion-mode OECTs, respectively. **b** Input and output given by the ternary inverter showing the three states “-1”, “0” and “1”. **c** Truth table of the ternary NOT gate.

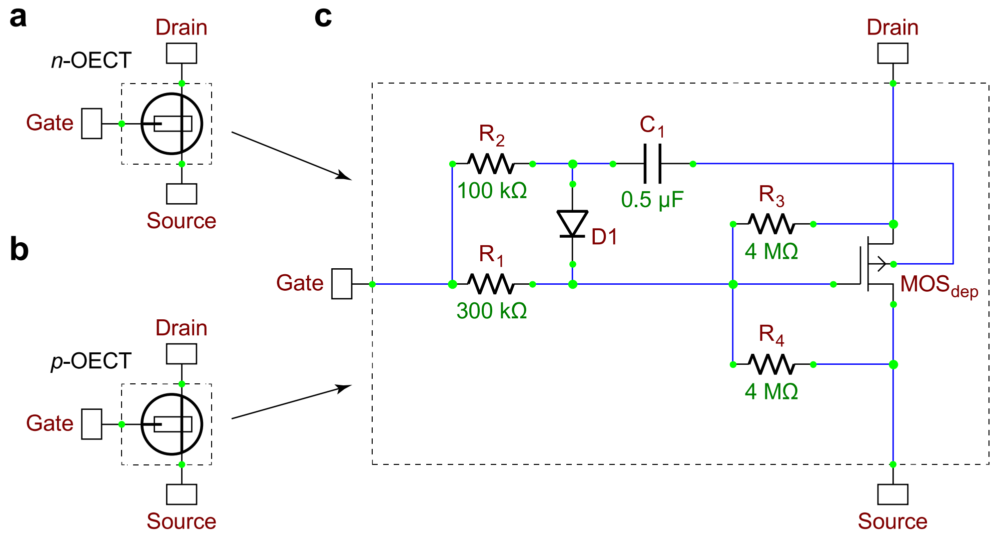

Supplementary Figure 36 | SPICE simulation models. a-b The symbols of *n*-type depletion-mode OECTs (BBL:PEI$_{lin}$, **a**) and *p*-type depletion-mode OECTs (PEDOT:PSS, **b**). **c** The SPICE models of both types are built as an equivalent sub-circuit, consisting of four resistors (R_1, R_2, R_3, R_4), one capacitor (C_1), one diode (D_1), and one depletion-mode MOS transistor (MOS_{dep}, level = 1, either *n*-type or *p*-type). R_1 = 300 kΩ, R_2 = 100 kΩ, C_1 = 0.5 μF, and D_1 are employed to model the asymmetric and slow switching of OECTs while R_3 = R_4 = 4 MΩ are used to model the leakage current from the gate. The device-to-device variation of both OECTs is modelled by the shift of the threshold voltage in the MOS transistor model.

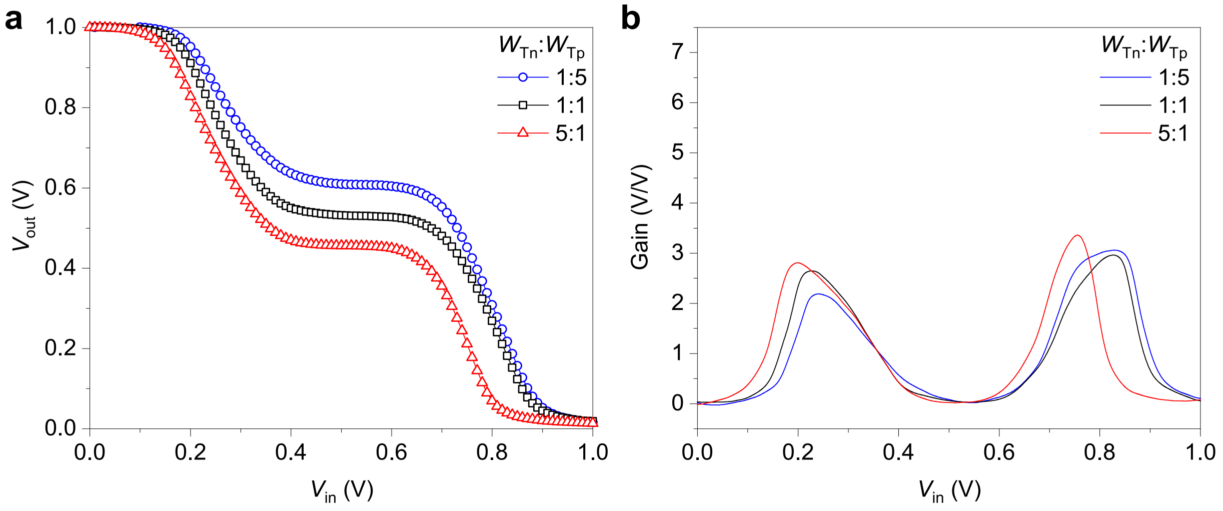

Supplementary Figure 37 | Comparison with silicon-based ternary inverters. a-b Transfer curve (**a**) and voltage gain (**b**) of a silicon-based tunnelling ternary inverter. Output voltage vs input voltage curves of silicon-based ternary inverter and corresponding gain reaching a maximum of 3. The transfer curves are extracted from Ref. [13], and the relative voltage gain is calculated by the first order differential. Data in (**a**) adapted with permission from Ref. [13]. Copyright 2019, The Author(s), under exclusive licence to Springer Nature Limited.

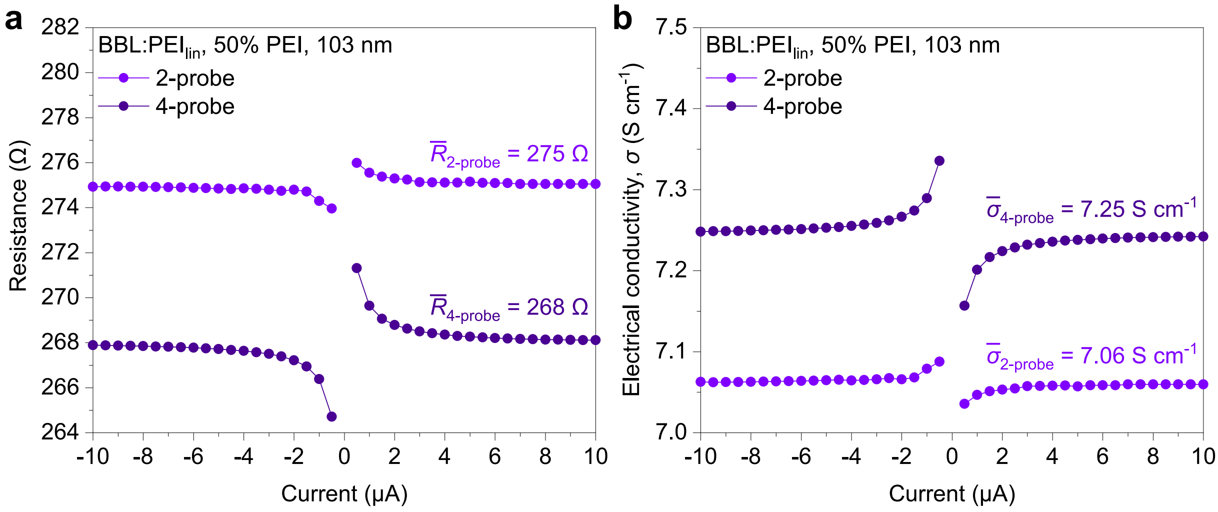

Supplementary Figure 38 | Resistance measurement method. a-b Resistance (**a**) and electrical conductivity (**b**) of BBL:PEI_{lin} measured by 4-probe and 2-probe method on the same film (channel width = 5 mm, channel length = 0.1 mm, gold electrode line-width of 80 µm, and film thickness of 103 nm). The resistance obtained with the 2-probe method is only 2.7 % higher than that obtained with the 4-probe method.

Supplementary Tables 1-6

Supplementary Table 1. Reported solution-processed n-type conducting polymers, electrical conductivity (σ), thermoelectric power factor (*PF*), and processing solvents. Regarding the solvent abbreviations, TCE = trichloroethylene, CB = chlorobenzene, ODCB = 1,2-dichlorobenzene, CF = chloroform, MSA = methanesulfonic acid, and Tol = toluene.

Materials	Year	σ (S cm^{-1})	*PF* (μW m^{-1}K^{-2})	Solvent	Reference
BBL:PEI	2020	7.7	11	Ethanol	This work
FBDPPV:TAM	2020	21	51	TCE/ODCB	[5]
PClClTVT:N-DMBI	2020	38.3	22.7	ODCB	[14]
PFClTVT:N-DMBI	2020	16.1	7.6	ODCB	[14]
PClClTVT:$CoCp_2$	2020	12.4	39.5	ODCB	[14]
PFClTVT:$CoCp_2$	2020	2.4	8.0	ODCB	[14]
BBL:P($g_4$2T-T)	2020	2	2.2	MSA	[15]
P(NDI2OD-T2):PEI	2020	2×10^{-3}	N.A.	CF	[16]
UFBDPPV:TAM	2020	22.5	80	ODCB	[17]
UFBDPPV:N-DMBI	2020	16.0	11.6	ODCB	[17]
LPPV-1:TAM	2020	3.7	33.9	ODCB	[17]
LPPV-1:N-DMBI	2020	0.9	1.7	ODCB	[17]
P(PzDPP-CT2):N-DMBI	2019	8.4	57.3	ODCB	[18]
FBDPPV:$(RuCp^*mes)_2$	2019	1.6	1	Tol	[19]
FBDPPV:N-DMBI	2019	7.1	4.8	Tol	[19]
FBDPPV:$(N\text{-}DMBI)_2$	2019	7.9	7	Tol	[19]
LPPV-1:N-DMBI	2019	1.10	1.96	ODCB/TCE	[20]
LPPV-2:N-DMBI	2019	0.07	0.25	ODCB/TCE	[20]
PDTzTI:TDAE	2019	4.6	7.6	CB	[21]
PDPF:N-DMBI	2018	1.3	4.65	ODCB	[22]
P(NDI2OD-Tz2):TDAE	2018	0.1	1.5	CF	[23]
P(NDI-alt-[$T2_{70}$-co-TPT_{30}]	2018	4×10^{-4}	N.A.	CB	[24]
2Strans-PNDIT2:N-DPBI	2018	6×10^{-3}	4.9×10^{-2}	Tol	[25]
PNDI2TEG-2Tz:N-DMBI	2018	1.8	4.6	CF	[26]
TEG-N2200:N-DMBI	2018	0.17	0.4	CF	[27]
p(gNDI-gT2):N-DMBI	2018	0.3	0.4	CF	[28]
ClBDPPV:TBAF	2017	0.62	0.63	CB	[29]
PNDTI-BBT-DT:N-DMBI	2017	0.18	0.6	CF	[30]
PNDTI-BBT-DP:N-DMBI	2017	5	14	CF	[30]
P(BTP-DPP): $(RuCp^*mes)_2$	2017	0.45	N.A.	CB/ODCB/Tol	[31]
P(PDI2OD-A):$(2\text{-}Cyc\text{-}DMBI)_2$	2016	0.27	N.A.	CB	[32]
P(PDI2OD-DEBT):$(2\text{-}Cyc\text{-}DMBI)_2$	2016	0.06	N.A.	CB	[32]
P(PDI2OD-T2):$(2\text{-}Cyc\text{-}DMBI)_2$	2016	0.0006	N.A.	CB	[32]
P(PDI2OD-E):$(2\text{-}Cyc\text{-}DMBI)_2$	2016	0.002	N.A.	CB	[32]
P(NDI2OD-T2):$(2\text{-}Cyc\text{-}DMBI)_2$	2016	0.003	N.A.	CB	[32]
BDPPV:N-DMBI	2015	0.26	2.5	ODCB	[4]
ClBDPPV:N-DMBI	2015	4	14	ODCB	[4]
FBDPPV:N-DMBI	2015	14	28	ODCB	[4]
P(NDIOD-T2):N-DMBI	2014	8×10^{-3}	0.6	ODCB	[33]
P(NDIOD-T2):N-DPBI	2014	4×10^{-3}	0.2	ODCB	[33]
P(NDI2OD-T2):2-Cyc-DMBI-H	2014	3.4×10^{-4}	N.A.	ODCB	[34]
P(NDI2OD-T2):$(2\text{-}Cyc\text{-}DMBI)_2$	2014	2.8×10^{-3}	N.A.	ODCB	[34]
P(NDI2OD-T2):$(2\text{-}Fc\text{-}DMBI)_2$	2014	7.6×10^{-5}	N.A.	ODCB	[34]
P(NDI2OD-T2):$(2\text{-}Rc\text{-}DMBI)_2$	2014	3.0×10^{-3}	N.A.	ODCB	[34]

Supplementary Table 2. Permissible Exposure Limit (PEL) and Immediately Dangerous to Life or Health (IDLH) for ethanol and aromatic/halogenated solvents typically used to process n-doped conducting polymers in Supplementary Table 1. Solvent abbreviation: Tol = toluene, CF = chloroform, TCE = trichloroethylene, CB = chlorobenzene, and ODCB = 1,2-dichlorobenzene. Data from the US National Institute for Occupational Safety and Health (NIOSH, www.cdc.gov/niosh/).

Solvents	Permissible exposure limit (ppm)	Immediately dangerous to life or health (ppm)
Ethanol	1000	3300
Tol	200	500
CF	50	500
TCE	100	1000
CB	75	1000
ODCB	50	200
MSA	N.A.	N.A.

Supplementary Table 3. Thickness of BBL:PEI_{lin} (50% PEI) films before and after wash with solvent in N_2 (unless specified otherwise). Solvent abbreviation: CB = chlorobenzene, CF = chloroform, DIO = 1,8-diiodooctane, DMF = dimethylformamide, DMSO = dimethylsulfoxide.

Solvents	Thickness before washing (nm)	Thickness after washing (nm)
CB	103.4±2.9	106.5±5.6
CF	102.2±5.4	107.3±3.5
DIO	99.1±2.6	102.1±4.7
DMF	107.3±4.3	104.2±2.4
DMSO	100.8±5.8	90.9±4.2
H_2O (air)	97.3±4.1	69.1±3.0

Supplementary Table 4. Absolute spin counts and spin densities in BBL and BBL:PEI films from EPR measurements.

Films	Abs. spin ($\times 10^{13}$ counts)	Volume ($\times 10^{-3}$ mm^{-3})	Spin density ($\times 10^{19}$ cm^{-3})
BBL	0.168	1.79	0.0941
BBL:PEI_{lin}, 5% PEI	2.11	2.11	1.00
BBL:PEI_{lin}, 20% PEI	10.7	2.24	4.77
BBL:PEI_{lin}, 50% PEI	19.4	2.56	7.58
BBL:PEI_{bra}, 5% PEI	2.06	2.15	0.958
BBL:PEI_{bra}, 20% PEI	19.0	2.25	8.46
BBL:PEI_{bra}, 50% PEI	105	3.58	29.3

Supplementary Table 5. Comparison of in-plane geometry polymer thermoelectric generator: thermoelectric leg materials, thermo module units (one unit = a pair of p and n legs), working temperature difference (ΔT), power output (P_{output}), and power output per p-n pair.

p-leg material	n-leg material	p-n pair	ΔT (K)	P_{output} (nW)	P_{output} (per p-n pair, nW)	Reference
PEDOT:PSS (gold electrodes)	BBL:PEI$_{lin}$	1	5	0.51	0.51	This work
			10	2.08	2.08	
			20	8.26	8.26	
			30	18.71	18.71	
			40	33.88	33.88	
			50	55.34	55.34	
PEDOT:PSS (silver electrodes)	BBL:PEI$_{lin}$	1	19.2	5.33	5.33	This work
			37.9	22.31	22.31	
			56.5	54.76	54.76	
PEDOT:PSS (gold electrodes)	FBDPPV:TAM	3	2.5	0.18	0.06	[5]
			4.3	0.51	0.17	
			9.8	2.75	0.92	
			17.5	9.24	3.08	
			27.8	24.75	8.25	
			46.5	77.02	25.67	
PEDOT:PSS	silver connection	1	65	0.057	0.057	[9]
PEDOT:PSS	silver connection	5	75.2	12.29	2.46	[10]
PEDOT:PSS	silver connection	385	50	4000	10.39	[11]
		1650	100	50000	30.30	
PEDOT:PSS	gold connection	10	10	2.6	0.26	[12]
			20	11	1.10	
			30	25	2.50	

Supplementary Table 6. List of materials developed for OECTs and their characteristics from literature. Carrier type, operation mode, transconductance (g_m) and typical working voltages (V_D and V_G) of state-of-the-art OECT materials.

Materials	Carrier type	Operation mode	g_m (mS)	V_D (V)	V_G (V)	Reference
PEDOT:PSS	p	depletion	2.5	-0.6	1.1	[35]
PEDOT:Tos	p	depletion	0.768	-0.2	0.5	[36]
PEDOT: PSTFSILi100	p	depletion	3.41	-0.8	0.8	[37]
PEDOT-S	p	depletion	15	-1.0	1.0	[38]
PEDOT-S:(Nonyl)NH_3	p	depletion	6.4	-0.4	0.4	[38]
PEDOT-S:$(Oct)_2NH_2$	p	depletion	8.3	-0.4	0.4	[38]
PPy	p	accumulation	N.A.	-0.2	-0.6	[39]
PANI	p	accumulation	0.45	-0.2	-0.3	[40]
Polycarbazole	p	accumulation	N.A.	-1.0	-0.9	[41]
P3MT	p	accumulation	6	-0.2	-1	[42]
P3HT	p	accumulation	0.04	-0.5	-1.2	[43]
P3MEEMT	p	accumulation	4.5	-0.7	-0.7	[44]
PTHS	p	accumulation	2.0	-0.6	-0.8	[45]
PTEB-S	p	accumulation	0.7	-0.25	-0.6	[46]
g2T-T	p	accumulation	7.9	-0.4	-0.7	[47]
gBDT-g2T	p	accumulation	0.47	-0.4	-0.75	[47]
gBDT	p	accumulation	1.2×10^{-4}	-0.4	-0.75	[47]
gBDT-T	p	accumulation	5.5×10^{-4}	-0.4	-0.7	[47]
gBDT-2T	p	accumulation	6.0×10^{-2}	-0.4	-0.7	[47]
gBDT-MeOT2	p	accumulation	1.82	-0.6	-0.6	[48]
gBDT-TT	p	accumulation	0.09	-0.4	-0.7	[48]
gBDT-T2	p	accumulation	0.06	-0.4	-0.65	[48]
p(a2T-TT)	p	accumulation	N.A.	-0.6	-0.6	[49]
p(g2T-TT)	p	accumulation	27	-0.6	-0.6	[49]
ProDOT(OE)-DMP	p	accumulation	0.622	-0.5	-0.8	[50]
PIBET-AO	p	accumulation	12.4	-0.6	-0.8	[51]
PIBET-O	p	accumulation	10.6	-0.6	-0.8	[51]
PIBET-BO	p	accumulation	5.1	-0.6	-0.9	[51]
PIBT-BO	p	accumulation	11.7	-0.6	-1	[51]
PIBET-A	p	accumulation	3.8	-0.6	-1	[51]
P3CPT	p	accumulation	26	-0.6	-1	[52]
p(gNDI-gT2)	n	accumulation	0.0217	0.6	0.55	[53]
P-100	n	accumulation	0.014	0.6	0.6	[54]
P-90	n	accumulation	0.047	0.6	0.6	[54]
P-75	n	accumulation	0.014	0.6	0.6	[54]
P-50	n	accumulation	0.005	0.6	0.6	[54]
BBL	n	accumulation	9.7	0.8	0.7	[55]
BBL:PEI	n	depletion	0.38	0.3	-0.7	This work

Supplementary References

1 Chua, L. L. *et al.* General observation of n-type field-effect behaviour in organic semiconductors. *Nature* **434**, 194-199 (2005).
2 Un, H. I. *et al.* Charge-trapping-induced non-ideal behaviors in organic field-effect transistors. *Adv. Mater.* **30**, 1800017 (2018).
3 Su, W. in *Printed Electronics: Materials, Technologies and Applications* (ed Zheng Cui) Ch. 8, (Wiley/Higher Education Press, 2016).
4 Shi, K. *et al.* Toward high performance n-type thermoelectric materials by rational modification of BDPPV backbones. *J. Am. Chem. Soc.* **137**, 6979-6982 (2015).
5 Yang, C.-Y. *et al.* A thermally activated and highly miscible dopant for n-type organic thermoelectrics. *Nat. Commum.* **11**, 3292 (2020).
6 Abragam, A. & Bleaney, B. *Electron paramagnetic resonance of transition ions*. (Oxford University Press, Oxford, 1970).
7 Kanemoto, K., Muramatsu, K., Baba, M. & Yamauchi, J. Polaron dynamics of heavily doped regioregular and regiorandom poly(3-alkylthiophenes) revealed by electron spin resonance spectroscopy. *J. Phys. Chem. B* **112**, 10922 (2008).
8 Krinichnyi, V. I. 2-mm Waveband electron paramagnetic resonance spectroscopy of conducting polymers. *Synth. Met.* **108**, 173-222 (2000).
9 Søndergaard, R. R., Hösel, M., Espinosa, N., Jørgensen, M. & Krebs, F. C. Practical evaluation of organic polymer thermoelectrics by large-area R2R processing on flexible substrates. *Energy Sci. Eng.* **1**, 81-88 (2013).
10 Du, Y. *et al.* Thermoelectric fabrics: toward power generating clothing. *Sci. Rep.* **5**, 6411 (2015).
11 Wei, Q., Mukaida, M., Kirihara, K., Naitoh, Y. & Ishida, T. Polymer thermoelectric modules screen-printed on paper. *RSC Adv.* **4**, 28802-28806 (2014).
12 Kim, N. *et al.* Elastic conducting polymer composites in thermoelectric modules. *Nat. Commum.* **11**, 1424 (2020).
13 Jeong, J. W. *et al.* Tunnelling-based ternary metal–oxide–semiconductor technology. *Nat. Electron.* **2**, 307-312 (2019).
14 Han, J. *et al.* Dichlorinated dithienylethene-based copolymers for air-stable n-type conductivity and thermoelectricity. *Adv. Funct. Mater.* **30**, 2005901 (2020).
15 Xu, K. *et al.* Ground-state electron transfer in all-polymer donor-acceptor heterojunctions. *Nat. Mater.* **19**, 738–744 (2020).
16 Seidel, K. F. *et al.* Single-step formation of a low work function cathode interlayer and n-type bulk doping from semiconducting polymer/polyethylenimine blend solution. *ACS Appl. Mater. Interfaces* **12**, 28801-28807 (2020).
17 Lu, Y. *et al.* The critical role of dopant cations in electrical conductivity and thermoelectric performance of n-doped polymers. *J. Am. Chem. Soc.* **142**, 15340–15348 (2020).
18 Yan, X. W. *et al.* Pyrazine-flanked diketopyrrolopyrrole (DPP): a new polymer building block for high-performance n-type organic thermoelectrics. *J. Am. Chem. Soc.* **141**, 20215-20221 (2019).
19 Un, H. I. *et al.* Understanding the effects of molecular dopant on n-type organic thermoelectric properties. *Adv. Energy Mater.* **9**, 1900817 (2019).
20 Lu, Y. *et al.* Rigid coplanar polymers for stable n-type polymer thermoelectrics. *Angew. Chem. Int. Ed.* **58**, 11390-11394 (2019).
21 Liu, J. *et al.* Overcoming coulomb interaction improves free-charge generation and thermoelectric properties for n-doped conjugated polymers. *ACS Energy Lett.* **4**, 1556-1564 (2019).
22 Yang, C. Y. *et al.* Enhancing the n-type conductivity and thermoelectric performance of donor-acceptor copolymers through donor engineering. *Adv. Mater.* **30**, 1802850 (2018).
23 Wang, S. *et al.* A chemically doped naphthalenediimide-bithiazole polymer for n-type organic thermoelectrics. *Adv. Mater.* **30**, 1801898 (2018).
24 Shin, Y. *et al.* Improving miscibility of a naphthalene diimide-bithiophene copolymer with n-Type dopants through the incorporation of "kinked" monomers. *Adv. Elec. Mater.* **4**, 1700581 (2018).
25 Nava, D. *et al.* Drastic improvement of air stability in an n-type doped naphthalene-diimide polymer by thionation. *ACS Appl. Energy Mater.* **1**, 4626-4634 (2018).

26 Liu, J. *et al.* N-type organic thermoelectrics of donor-acceptor copolymers: improved power factor by molecular tailoring of the density of states. *Adv. Mater.* **30**, 1804290 (2018).
27 Liu, J. *et al.* Enhancing molecular n-type doping of donor-acceptor copolymers by tailoring side chains. *Adv. Mater.* **30**, 1704630 (2018).
28 Kiefer, D. *et al.* Enhanced n-doping efficiency of a naphthalenediimide-based copolymer through polar side chains for organic thermoelectrics. *ACS Energy Lett.* **3**, 278-285 (2018).
29 Zhao, X. *et al.* High conductivity and electron-transfer validation in an n-type fluoride-anion-doped polymer for thermoelectrics in air. *Adv. Mater.* **29**, 1606928 (2017).
30 Wang, Y. *et al.* Naphthodithiophenediimide-benzobisthiadiazole-based polymers: versatile n-type materials for field-effect transistors and thermoelectric devices. *Macromolecules* **50**, 857-864 (2017).
31 Perry, E. E. *et al.* High conductivity in a nonplanar n-doped ambipolar semiconducting polymer. *Chem. Mater.* **22**, 9742-9750 (2017).
32 Naab, B. D. *et al.* Role of polymer structure on the conductivity of n-doped polymers. *Adv. Elec. Mater.* **2**, 1600004 (2016).
33 Schlitz, R. A. *et al.* Solubility-limited extrinsic n-type doping of a high electron mobility polymer for thermoelectric applications. *Adv. Mater.* **26**, 2825-2830 (2014).
34 Naab, B. D. *et al.* Effective solution- and vacuum-processed n-doping by dimers of benzimidazoline radicals. *Adv. Mater.* **26**, 4268-4272 (2014).
35 Ersman, P. A. *et al.* All-printed large-scale integrated circuits based on organic electrochemical transistors. *Nat. Commum.* **10**, 5053 (2019).
36 Jimison, L. H. *et al.* PEDOT:TOS with PEG: a biofunctional surface with improved electronic characteristics. *J. Mater. Chem.* **22**, 19498–19505 (2012).
37 Inal, S. *et al.* Organic electrochemical transistors based on PEDOT with different anionic polyelectrolyte dopants. *J. Polym. Sci. Polym. Phys.* **54**, 147-151 (2016).
38 Zeglio, E., Eriksson, J., Gabrielsson, R., Solin, N. & Inganas, O. Highly stable conjugated polyelectrolytes for water-based hybrid mode electrochemical transistors. *Adv. Mater.* **29**, 1605787 (2017).
39 White, H. S., Kittlesen, G. P. & Wrighton, M. S. Chemical derivatization of an array of 3 gold microelectrodes with polypyrrole - fabrication of a molecule-based transistor. *J. Am. Chem. Soc.* **106**, 5375-5377 (1984).
40 Paul, E. W., Ricco, A. J. & Wrighton, M. S. Resistance of polyaniline films as a function of electrochemical potential and the fabrication of polyaniline-based microelectronic devices. *J. Phys. Chem.* **89**, 1441-1447 (1985).
41 Rani, V. & Santhanam, K. S. V. Polycarbazole-based electrochemical transistor. *J. Solid State Electrochem.* **2**, 99-101 (1998).
42 Thackeray, J. W., White, H. S. & Wrighton, M. S. Poly(3-methylthiophene)-coated electrodes: optical and electrical properties as a function of redox potential and amplification of electrical and chemical signals using poly(3-methylthiophene)-based microelectrochemical transistors. *J. Phys. Chem.* **89**, 5133-5140 (1985).
43 Matsui, J., Sato, Y., Mikayama, T. & Miyashita, T. Fabrication of electrochemical transistor based on pi-conjugate polymer Langmuir-Blodgett film. *Langmuir* **23**, 8602-8606 (2007).
44 Flagg, L. Q. *et al.* Polymer crystallinity controls water uptake in glycol side-chain polymer organic electrochemical transistors. *J. Am. Chem. Soc.* **141**, 4345-4354 (2019).
45 Inal, S. *et al.* A High transconductance accumulation mode electrochemical transistor. *Adv. Mater.* **26**, 7450-7455 (2014).
46 Zeglio, E. *et al.* Conjugated polyelectrolyte blends for electrochromic and electrochemical transistor devices. *Chem. Mater.* **27**, 6385-6393 (2015).
47 Nielsen, C. B. *et al.* Molecular design of semiconducting polymers for high-performance organic electrochemical transistors. *J. Am. Chem. Soc.* **138**, 10252-10259 (2016).
48 Giovannitti, A. *et al.* Redox-stability of alkoxy-BDT copolymers and their use for organic bioelectronic devices. *Adv. Funct. Mater.* **28**, 1706325 (2018).
49 Giovannitti, A. *et al.* Controlling the mode of operation of organic transistors through side-chain engineering. *Proc. Natl. Acad. Sci. U.S.A.* **113**, 12017-12022 (2016).

50 Savagian, L. R. *et al.* Balancing charge storage and mobility in an oligo(ether) functionalized dioxythiophene copolymer for organic- and aqueous- based electrochemical devices and transistors. *Adv. Mater.* **30**, 1804647 (2018).

51 Wang, Y. *et al.* Hybrid alkyl–ethylene glycol side chains enhance substrate adhesion and operational stability in accumulation mode organic electrochemical transistors. *Chem. Mater.* **31**, 9797-9806 (2019).

52 Khau, B. V., Savagian, L. R., De Keersmaecker, M., Gonzalez, M. A. & Reichmanis, E. Carboxylic acid functionalization yields solvent-resistant organic electrochemical transistors. *ACS Mater. Lett.* **1**, 599-605 (2019).

53 Giovannitti, A. *et al.* N-type organic electrochemical transistors with stability in water. *Nat. Commun.* **7**, 13066 (2016).

54 Giovannitti, A. *et al.* The role of the side chain on the performance of n-type conjugated polymers in aqueous electrolytes. *Chem. Mater.* **30**, 2945-2953 (2018).

55 Sun, H. *et al.* Complementary logic circuits based on high-performance n-type organic electrochemical transistors. *Adv. Mater.* **30**, 1704916 (2018).
